# Oriented cell divisions induce basal progenitors and regulate neural expansion across tissues and species

**DOI:** 10.1126/sciadv.adz6827

**Published:** 2026-02-04

**Authors:** Benoit Boulan, Marine Lacomme, Amin Benadjal, Miranda Krueger, Ko Currie, Anna La Torre, Alain Chédotal, Michel Cayouette

**Affiliations:** ^1^ Cellular Neurobiology Research Unit, Institut de recherches cliniques de Montréal (IRCM), Montréal, Canada.; ^2^ Sorbonne Université, Inserm, CNRS, Institut de la Vision, 17 rue Moreau, F-75012 Paris, France.; ^3^ Department of Cell Biology and Human Anatomy, University of California Davis, Davis, CA, USA.; ^4^ Institut de pathologie groupe hospitalier Est, hospices civils de Lyon, Lyon, France.; ^5^ MeLiS, CNRS UMR5284, Inserm U1314, University Claude Bernard Lyon 1, Lyon, France.; ^6^ Department of Medicine, Université de Montréal, Montréal, Canada.; ^7^ Department of Anatomy and Cell Biology, McGill University, Montréal, Canada.

## Abstract

Nervous system expansion relies on progenitor proliferation, yet its regional and evolutionary regulation is incompletely understood. While basally dividing progenitors are implicated in neocortical growth, their developmental origins and relevance beyond the cortex remain unclear. We show here that double inactivation of spindle orientation regulators GPSM2 and SAPCD2 in mice completely reorients progenitor divisions in both the neocortex and retina. This shift increases basal progenitors over sixfold in the neocortex and induces their ectopic emergence in the retina, resulting in extra cell layers and ~30% tissue enlargement. Single-cell RNA sequencing reveals that the induced basal progenitors in the cortex resemble human outer radial glia, and both cortical and retinal progenitors show altered Hippo signaling. Last, macaque and human retinas display twice as many reoriented divisions as the mouse and naturally contain basal progenitors. These findings show that division orientation is critical for regulating neural progenitor output and scaling tissue growth.

## Teaser

A fundamental role for division orientation in progenitor output driving cortical and retinal growth is revealed.

## INTRODUCTION

The development of the central nervous system (CNS) requires precise regulation of neural progenitor cell proliferation and differentiation to generate tissues of accurate size and cellular composition. Any deviation from this meticulous process can result in severe neurodevelopmental disorders such as microcephaly, lissencephaly, and subcortical band heterotopia (*1*).

During mammalian corticogenesis, neural progenitors are broadly classified by their location and proliferation capacity. Apical progenitors, or radial glial cells (RGCs), divide at the ventricular surface of the neuroepithelium and can self-renew or give rise to intermediate progenitors (IPs), which divide more basally in the subventricular zone and typically undergo terminal neurogenic divisions (*2*). Outer RGCs (oRGCs) divide even further from the ventricular surface and have greater proliferative capacity (*3*). These oRGCs are disproportionately enriched in gyrencephalic species such as humans, ferrets, and sheep, supporting the model that they drive cortical expansion (*4*–*6*). Although several markers of oRGCs have been identified, the mechanisms governing their emergence remain poorly understood.

Recent work implicates the Hippo pathway, a key regulator of organ size, in IP production in developing mouse neocortex (*7*). However, how its effectors Yes-associated protein 1 (YAP1)/TAZ become activated in specific progenitor subtypes is unclear. One possibility is through the asymmetric partitioning of signaling regulators during mitosis, which depends on mitotic spindle orientation (*8*). In early mouse cortical development, RGCs predominantly divide with their mitotic spindle aligned parallel to the apical surface (horizontal), resulting in symmetric proliferative divisions (*8*). At later stages, some RGCs reorient their mitotic spindle along the apicobasal axis (vertical), resulting in asymmetric proliferative divisions that generate a RGC and an IP or an oRGC (*9*, *10*).

In contrast to the neocortex, the mammalian retina is thought to develop from a single class of apically dividing, multipotent retinal progenitors. While basal divisions have been reported in the chick and zebrafish retina, these divisions arise from fate-restricted neuronal precursors giving rise to neurons locally, and do not contribute to amplifying the progenitor pool (*11*–*14*). Basally dividing progenitors have not been reported in the mouse retina, but whether such cells might exist in species with larger retinas, and contribute to tissue growth, remains unknown. Mouse retinal progenitors share several characteristics with cortical RGCs, such as expression of *Pax6*, *Slc1a3* (Glast), and *Sox2* (*15*–*17*), as well as the ability to generate multiple cell types in a temporal sequence (*18*, *19*). Although apicobasal reorientation of the mitotic spindle has been shown to regulate late-stage asymmetric neurogenic divisions in the retina (*20*–*22*), its role during earlier proliferative phases of mammalian retinogenesis is still unknown.

Mitotic spindle orientation in mammalian neural progenitors is controlled by evolutionarily conserved proteins such as the Gαi–G protein signaling modulator 2 (GPSM2)–nuclear mitotic apparatus (NuMA) complex and the partitioning-defective (PAR) polarity complex (*23*). These complexes are modulated by various regulators including suppressor APC domain–containing 2 (SAPCD2), ASPM, AGS3, CITK, DLG1, EML1, and mINSC, which converge to have diverse context-dependent importance (*24*–*29*). However, loss-of-function studies of these regulators in mice have typically yielded mild changes in cell fates, prompting extensive and continuing debates in the field about the significance of division orientation in neurodevelopment (*30*–*33*). In contrast, mutants for apico-basal polarity determinants often display more severe tissue disorganization and fate changes, with widespread defects in adhesion and lamination (*34*). However, because these phenotypes affect multiple cellular processes beyond spindle orientation, including cell polarity and adhesion, it has been difficult to isolate the specific contribution of division orientation in tissue expansion.

To address this issue, we focused our attention on GPSM2 (also known as LGN or AGS3-like in vertebrates, or Pins in *Drosophila*) and SAPCD2 (also known as ang, p42.3, or C9orf140). Both GPSM2 and SAPCD2 are adaptor proteins that function to regulate the Gαi-GPSM2-NuMA spindle orientation complex. On the one hand, GPSM2 is a scaffolding protein containing TPR and GoLoco domains that binds NuMA (Mud in flies) and dynein to generate pulling forces on aster microtubules, thereby aligning the mitotic spindle with the cell polarity axis (*8*, *35*). On the other hand, SAPCD2 negatively regulates the cortical localization of GPSM2 to control its activity in the context of division orientation. In a previous study, we showed that SAPCD2 localizes apically in retinal progenitors, leading to lateral localization of GPSM2 and thus planar divisions (*24*). Given these complementary roles in the regulation of division orientation, we hypothesized that simultaneous inactivation of *Sapcd2* and *Gpsm2* would disrupt spindle orientation machinery specifically without affecting tissue polarity, thereby allowing us to study the role of division orientation in neural expansion. Notably, we report that dKO mice exhibit a complete inversion in the distribution of division orientations in both retinal and cortical tissues, leading to a marked increase in basally dividing progenitors in the neocortex and de novo emergence of basal progenitors in the retina. This eventually leads to tissue hyperplasia and the formation of additional cell layers in adult tissues. Single-cell RNA sequencing shows that dKO cortical basal progenitors resemble human oRGCs, and both the cortex and retina display altered Hippo signaling. Inactivation of the Hippo pathway is sufficient to induce basally dividing progenitors in the mouse retina and trigger overproduction of neurons. Last, we show that the larger macaque and human retinas exhibit more vertical divisions than the mouse and feature basal progenitors. These findings establish a critical and conserved role for division orientation in the production of basal progenitors in multiple neural tissues and highlight the importance of this process in regulating neural tissue size across species.

## RESULTS

### GPSM2 and SAPCD2 are essential for horizontal divisions

Although GPSM2 was previously studied in mouse cortical development (*35*), the role of SAPCD2 in the regulation of mitotic spindle orientation in the cortex remains unknown. To explore this question, we first used a published single-cell RNA sequencing (scRNA-seq) dataset of developing mouse cortices ranging from embryonic day (E) 10 to postnatal day (P) 4 to analyze *Sapcd2* expression. We found that *Sapcd2* is coexpressed with *Gpsm2* in apical RGCs, IP, and glial cells, with a peak of expression at around E12 (fig. S1, A to E). *Gpsm2* and *Sapcd2* expression is up-regulated during G2/M (fig. S1, F and G), consistent with previous studies (*24*, *36*), and with their role in regulating mitotic spindle orientation. As in retinal progenitors (*24*), we found that the SAPCD2 protein is enriched at the apical pole of RGCs (fig. S1H), consistent with a possible role in regulating RGC division orientation.

**Fig. 1.**
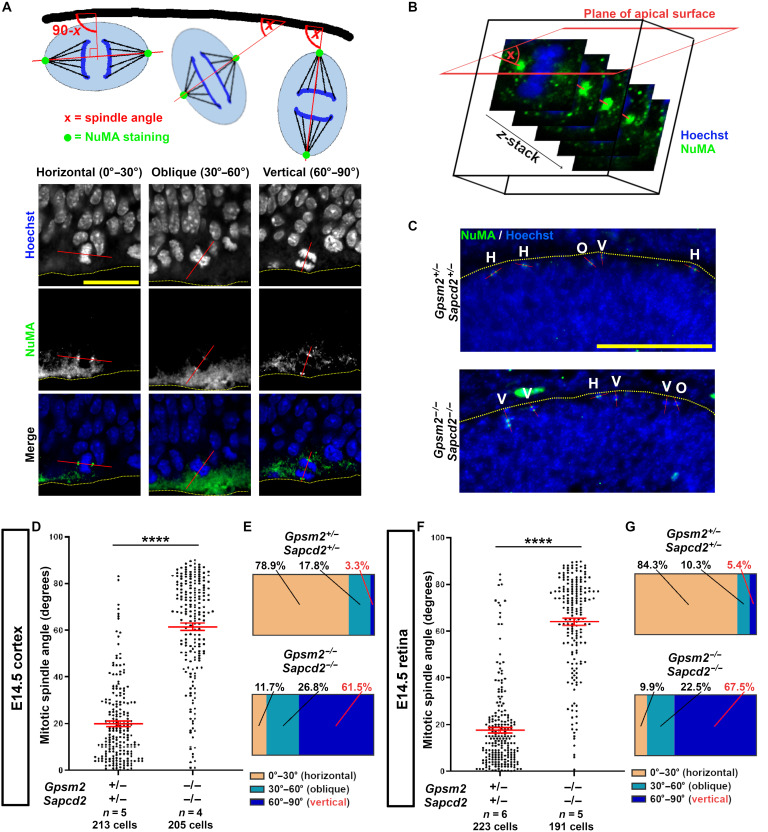
GPSM2 and SAPCD2 function synergistically to promote horizontal divisions. (**A**) Schematic (top) and representative confocal images (bottom) showing the method for mitotic spindle orientation quantification and definition of angle values. NuMA staining is used to define the position of sister centrioles. The thick black line on the schematic and dashed yellow bar line on microscopy images represent the apical surface of the neuroepithelium (retina or cortex), which is used as a reference. Scale bar, 20 μm. (**B**) 3D Illustration of mitotic spindle angle measurements. *z*-stacks of dividing progenitors are used to define the 3D orientation of the mitotic spindle relative to the plane of the apical surface of the tissue. (**C**) Representative image of NuMA immunostainings on a E14.5 retinal section. The dotted line outlines the apical surface, and the red line indicates the mitotic spindle orientation. Scale bar, 25 μm. (**D** to **G**) Quantification of progenitor mitotic spindle orientation in E14 cortex [(D) and (E)] and retina [(F) and (G)]. Each dot represents the orientation of a dividing progenitor. Total number of animals and cells analyzed is shown at the bottom of each graph. Red bars represent means ± SEM. Bar graphs show spindle angle relative frequency (%) segregated into three bins for horizontal (0° to 30°), oblique (30° to 60°), and vertical (60° to 90°) divisions. Comparisons between groups were done using nonparametric Mann-Whitney test (****P < 0.0001).

To study the role of *Gpsm2* and *Sapcd2* in cell division orientation, we established a new mouse line by first crossing *Gpsm2^+/−^* with *Sapcd2^−/−^* mice. Double heterozygous (dHET) offsprings from this new line were then intercrossed to produce dKO and control littermates. We then studied cell division orientation in cortical sections at E14.5. We stained for NuMA to label the centrioles and measured the orientation of the mitotic spindle relative to the plane of the neuroepithelium by tracing a line between the two centrioles in 3D (Fig. 1A). The resulting spindle angle *x* was classified as horizontal (0° to 30°), oblique (30° to 60°) or vertical (60° to 90°) (Fig. 1, A and B). In double heterozygotes, we found that most RGC divisions are horizontal (Fig. 1, D and E), comparable to the WT cortex (fig. S2A), whereas single KO of either *Gpsm2* or *Sapcd2* on a heterozygous background for *Sapcd2* or *Gpsm2*, respectively, show a significant increase in oblique and vertical divisions (fig. S2A). These results recapitulate previous observations in *Gpsm2* KO (*35*), and additionally show that *Sacpd2* is required for proper division orientations in the developing neocortex, as previously reported in the retina (*24*). More notably, however, we observed that dKO mice have considerably more oblique and vertical divisions than single KOs in the developing neocortex (fig. S2A), with an inversion in the distribution of RGC division orientations compared to controls (Fig. 1, D and E). These results show that *Sapcd2* and *Gpsm2* genetically interact to regulate cortical progenitor cell division orientation, and that both genes are required to maintain most divisions in the horizontal plane.

Previous studies from our group demonstrated that GPSM2 promotes vertical divisions in the retina (*22*). In contrast, SAPCD2 promotes horizontal divisions in the retina (*24*). The above results show that single inactivation of *Sapcd2* increases vertical divisions in the cortex, paralleling its function in the retina, while combined inactivation of *Sapcd2* and *Gpsm2* produces a marked increase in vertical divisions in the cortex. Given the shared developmental origin of the retina and cortex, these findings suggested that the two adaptor proteins might operate in a comparable regulatory framework across neuroepithelia. We therefore hypothesized that double inactivation in the retina would yield a similar phenotype. As previously reported (*37*), we found that WT retinas at E14.5 have less than 10% of combined oblique and vertical divisions, whereas double heterozygotes (*Gpsm2^+/−^;Sapcd2^+/−^*) show a slight increase to ~16% (fig. S2B). In single KOs of *Gpsm2* or *Sapcd2* on a heterozygote background of *Sapcd2* or *Gpsm2*, respectively, we found a more important increase in the proportion of oblique/vertical divisions (fig. S2B). Most notably, however, we observed ~90% oblique/vertical divisions in dKO retinas, a near-complete inversion in the distribution of division orientations compared to double heterozygote controls (Fig. 1, C, F, and G). These results show that *Gpsm2* and *Sapcd2* are partially redundant to promote horizontal progenitor cell divisions during early stages of retinal development.

### Cell division orientation drives basal progenitor cell production and proliferation in both the cortex and retina

**Fig. 2.**
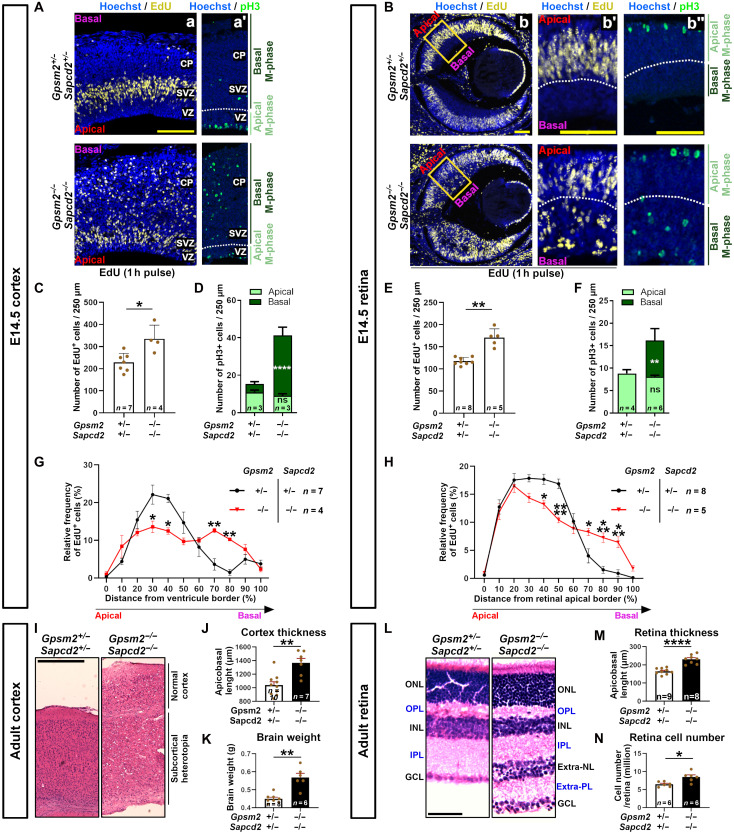
Inactivation of *Gpsm2* and *Sapcd2* increases the number of neural progenitor cells, basal divisions, and adult tissue size. (**A** and **B**) EdU labeling (yellow) after a 1-hour pulse (a, b, and b′) and phospho–histone-3 (pH3) immunostaining (green) (a′ and b″) on E14.5 cortical (A) and retinal (B) sections from dHET (*Gpsm2^+/−^; Sapcd2^+/−^*) and dKO (*Gpsm2^−/−^; Sapcd2^−/−^*) mice. Zoom in of regions identified in subpanel b is shown in subpanel b′. Scale bars: 100 μm. (**C** to **F**) Quantification of the total number of EdU^+^ and pH3^+^ progenitors in E14.5 cortex [(C) and (D)] and retina [(E) and (F)]. (**G** and **H**) Quantification of EdU^+^ cell localization relative to the apical surface of cortical (G) and retinal sections (H). Distance between EdU^+^ cells and apical border of the tissue is measured and expressed as a percentage of the tissue thickness. (**I**) Hematoxylin and eosin coloration of dHET and dKO sections of adult cortex. Scale bar, 500 μm. (**J** and **K**) Quantification of cortical tissue thickness (J) and adult brain weight in dHET and dKO (K). (**L**) Hematoxylin and eosin coloration of dHET and dKO sections of adult retina. Scale bar, 50 μm. (**M** and **N**) Quantification of retinal thickness (M) and total cell numbers (N) in dHET and dKO adult retina. Comparison of EdU cell localization and pH3 cell count was done using a two-way analysis (ANOVA) test followed by Sidak’s correction for multiple comparisons. All other comparisons between dHET and dKO samples were done using Mann Whitney tests. (*P < 0.05, **P < 0.01, ***P < 0.001, and ****P < 0.0001). ONL, outer nuclear layer; INL, inner nuclear layer; GCL, ganglion cell layer; OPL, outer plexiform layer; IPL, inner plexiform layer; extra-NL, extra nuclear layer; extra-PL, extra plexiform layer.

Previous studies have shown that an increase in vertical divisions correlates with an expansion of the basal progenitor pool in the developing mouse neocortex (*22*, *35*, *38*). To determine whether basal progenitors are increased in dKO embryos, we performed a 1-hour pulse of EdU at E14.5 and then stained cortical sections for phosphorylated histone H3 (pH3) and EdU to label mitotic and S-phase cells, respectively (Fig. 2A). In the dKO cortex, we observed an increase in the total number of EdU^+^ cells (Fig. 2C), originating from an increase in the number of EdU^+^ cells in the subventricular zone at the expense of apically located cells (Fig. 2G). The increase in basal EdU^+^ cells was less important in the E14.5 cortex of *Gpsm2^−/−^; Sapcd2^+/−^* and *Gpsm2^+/−^; Sapcd2^−/−^* embryos (fig. S2E, blue and purple lines). In addition, we found a more than sixfold increase in the number of basally located pH3^+^ cells in the dKO cortex compared to dHET (Fig. 2, A and D). These results indicate that GPSM2 and SAPCD2 together negatively control the number of basal progenitors in the developing neocortex.

In the developing mouse retina, basal progenitors have not been reported and the rare vertical divisions observed perinatally were shown to control the production of asymmetric neurogenic divisions, rather than proliferative asymmetric divisions (*20*, *21*, *37*). However, the drastic increase in oblique/vertical divisions observed in the dKO retina from early embryonic stages (Fig. 1F) offered a unique opportunity to determine whether reorientation of cell division is sufficient to induce basal progenitor production in a tissue that does not normally have any, and if so, whether these basal progenitors drive over proliferation. Notably, we found over proliferation in the E14.5 dKO retina, as observed in the cortex (Fig. 2, B, E, and F), primarily due to an increase in EdU^+^ and pH3^+^ cells the basal part of the retinal neuroepithelium (Fig. 2, F and H).

The basal divisions were not the result of a loss of tissue polarity, as we found no changes in the localization of apical markers PAR3, PODXL, or ZO1 in dKO retina and cortex compared to dHET (fig. S3, A and B), and immunoprecipitation of PAR3 followed by mass spectrometry (IP-MS) revealed no differences in the PAR3 interactome between dKO and dHET samples from either the retina or cortex at P0 (fig. S3, C and D, and table S1). Last, cell death was only minimally increased in the cortex and not changed in the retina at E14.5 (fig. S4), ruling out that reoriented divisions and overproduction of basal progenitors cause massive cell death.

We next asked whether basally dividing progenitors have a distinct morphology than classical retinal neuroepithelial cells dividing at the apical surface. While neuroepithelial cells maintain apical and basal attachments during the entire cell cycle (*20*, *39*), a classical feature of cortical basal progenitors is their lack of apical process (*40*). To characterize the morphology of ectopic basal progenitors generated in dKO retina, we stained retinal sections for phospho-vimentin, a maker of neural progenitors that clearly delineates the entire cell outline. We found cells on the basal side of the neuroepithelium that have lost their apical process and display a unipolar morphology typical of basal progenitors (fig. S5). Thus, excess vertical divisions promote basal progenitor production and over proliferation in both the cortex and retina.

### Basal progenitor cells drive neural tissue expansion in the cortex and retina

On the basis of the increased number of progenitor cells and elevated frequency of basal divisions observed in dKO retina and cortex, we postulated that this may lead to hyperplasia in adult tissues. As predicted, we found that the cortex (Fig. 2, I and J) and retina (Fig. 2, L and M) were thicker in dKO compared to dHET controls. Notably, the dKO retina contained an additional cell layer located between the inner nuclear layer (INL) and the ganglion cell layer (GCL), as well as an additional plexiform layer (Fig. 2L). In the cortex, we found subcortical heterotopia characterized by a medio-dorsal subventricular mass of gray matter (fig. S6A, white stars) surrounded by a white matter capsule (fig. S6A, yellow arrowheads). The increase in thickness was not simply caused by increased intercellular space or tissue stretching, as the dKO brain was heavier and the retina contained more cells than controls (Fig. 2, K and N). We also noticed variability in the phenotypes, with occurrences of hemorrhagic hydrocephalus in the worst cases (fig. S6B), and a range in severity of subcortical heterotopia, cortex size, and brain weight (Fig. 2, J and K, and fig. S6A).

To assess basal progenitor proliferation more specifically, we performed EdU/BrdU double-pulse by administering EdU for 24 hours at E14.5 followed by a 1-hour BrdU pulse before tissue collection and labelling. Approximately 15% of EdU^+^ dKO progenitors outside the cortical VZ/SVZ were also BrdU^+^, indicating continued cycling (fig. S7, A and C). In the dKO retina, where progenitors cycle faster than RGCs, this fraction increased to ~40% (fig. S7, B and D). Thus, despite their relatively low abundance, the ability of most retinal basal progenitors to undergo successive divisions likely contributes to the increased retinal thickness at late developmental stages. Together, these results suggest that progenitor cell division orientation ultimately regulates neural tissue size.

### Reoriented divisions generate specific cell types in both the cortex and retina

**Fig. 3.**
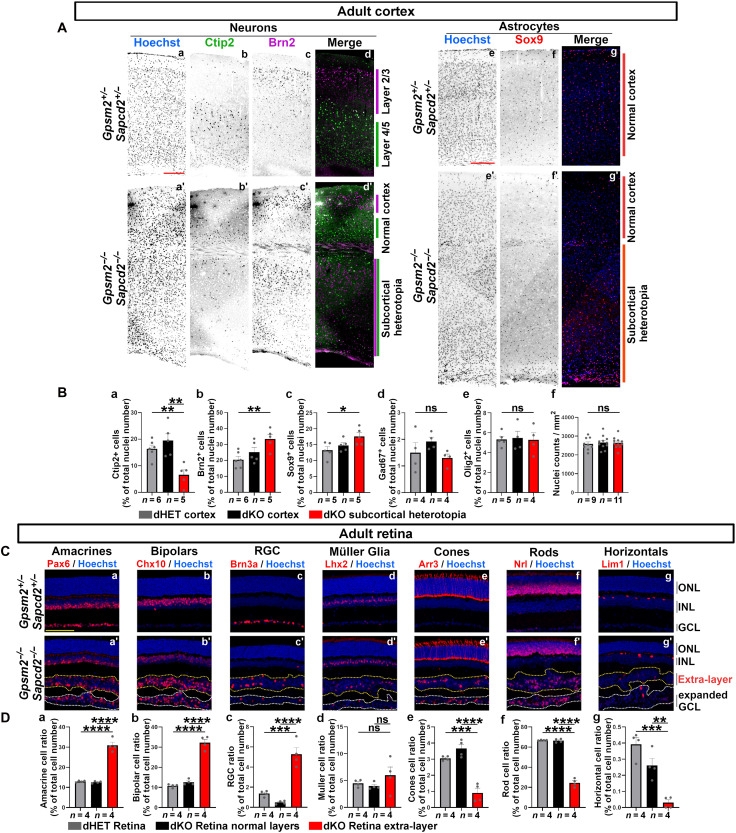
Cell type–specific increase in dKO retina and cortex. (**A**) Representative images of adult cortical sections from dHET and dKO stained for markers of upper layer II/III neurons (BRN2), deeper layers IV/V neurons (CTIP2), and astrocytes (SOX9). Scale bar, 200 μm. (**B**) Quantification of cell type proportions in adult dHET cortex, dKO cortex, and dKO subcortical heterotopia band. Upper layer II/III, deeper layer IV/V neurons, astrocytes, interneurons, and oligodendrocytes cell types are identified using BRN2/Pou3f2, CTIP2, SOX9, GAD67, and OLIG2, respectively. (**C**) Representative images of adult retinal sections from dHET and dKO stained for markers of amacrines (PAX6), bipolars (CHX10), retinal ganglion cells (BRN3a/POU4F1), Müller cells (LHX2), cones (ARR3), rods (NRL), and horizontals (LIM1). Yellow dashed lines underlie the extra retinal layer of dKO retina, white dashed lines underlie the expanded ganglion cell layer of dKO retina. Scale bar, 100 μm. (**D**) Quantification of retinal cell type proportions in adult dHET and dKO retina (excluding the extra layer, gray and black bars) and in the extra layer (red bars). Error bars represent mean ± SEM. All comparisons between groups were done using a one-way ANOVA test followed by Tukey’s correction for multiple comparisons. (*P < 0.05, **P < 0.01, ***P < 0.001, and ****P < 0.0001).

To investigate the resulting cellular composition and organization of neural tissues in absence of *Sapcd2* and *Gpsm2*, we stained the adult neocortex (Fig. 3A) and retina (Fig. 3C) for various cell type-specific markers. We found that, in addition to an apparently normal-layered cortex, a subcortical heterotopic layer arises, composed of various neuron types intermingled together (Fig. 3, A and B). Similarly, in the retina, an additional layer containing all major retinal cell types formed between the INL and GCL (Fig. 3, C and D). In the dKO cortex, the subcortical heterotopic layer contained more BRN2 (POU3F2)-positive upper-layer neurons (Fig. 3Bb) and SOX9-positive astrocytes (Fig. 3Bc), and fewer CTIP2-positive deep layer neurons (Fig. 3Ba), whereas inhibitory GABAergic neurons (GAD67) and oligodendrocytes (OLIG2) were unchanged compared to dHET controls (Fig. 3B, b and e). In addition, total cell density was unchanged in the different layers of the dKO (Fig. 3Bf), although the laminar organization of dKO neocortex was disrupted in the subcortical heterotopic layer but normal in the cortex above (Fig. 3A). These results suggest that vertical divisions tend to give rise to later-born superficial layer neurons and astrocytes in the cortex.

In the retina, amacrine, bipolar, and retinal ganglion cells were over-represented in the extra layer (Fig. 3D, a to c), Müller glia were unchanged (Fig. 3Dd), whereas rod and cone photoreceptors as well as horizontal cells were underrepresented (Fig. 3D, e to g). In addition, we observed all retinal cell types in the expanded ganglion cell layer of dKO (Fig. 3, C and D). Thus, the general organization of the basal cell layers of the dKO retina is severely disrupted with the presence of numerous cell types in the ganglion cell layer and the formation of an ectopic layer, whereas the other layers are normal.

Retinal cell subtypes are produced in a temporally controlled sequence, prompting us to explore whether the changes in cell type proportions observed in dKO animals are due to altered timing of cell type generation. To do this, we injected EdU either at early (E14.5) or late (P0) stages of retina development and assessed the timing of various cell type production (fig. S8) at P21. Costaining for CHX10 and EdU on animals that received EdU at E14.5 revealed an increase in production of late-born bipolar cells (CHX10^+^ EdU^+^) in both the INL and the extra-layer of dKO retinas (fig. S8, A and B), suggesting that bipolar cells in both the INL and ectopic layer are produced prematurely in dKO retinas. Similarly, costaining for PAX6 and EdU on animals that received EdU at P0 revealed an increase in early-born amacrine cells (PAX6^+^ EdU^+^) in dKO (fig. S8, C and D), suggesting an extension of the amacrine cell production window (or retinal ganglion cells, which also express PAX6). Together, these results show that the excess number of reoriented divisions and the resulting basal progenitors in dKO disrupts spatiotemporal patterning of neurogenesis, leading to premature production of late-born cell types and prolonged generation of early-born neurons.

### scRNA-seq reveals overproduction of outer RGCs in the *Gpsm2/Sapcd2* dKO mouse neocortex

**Fig. 4.**
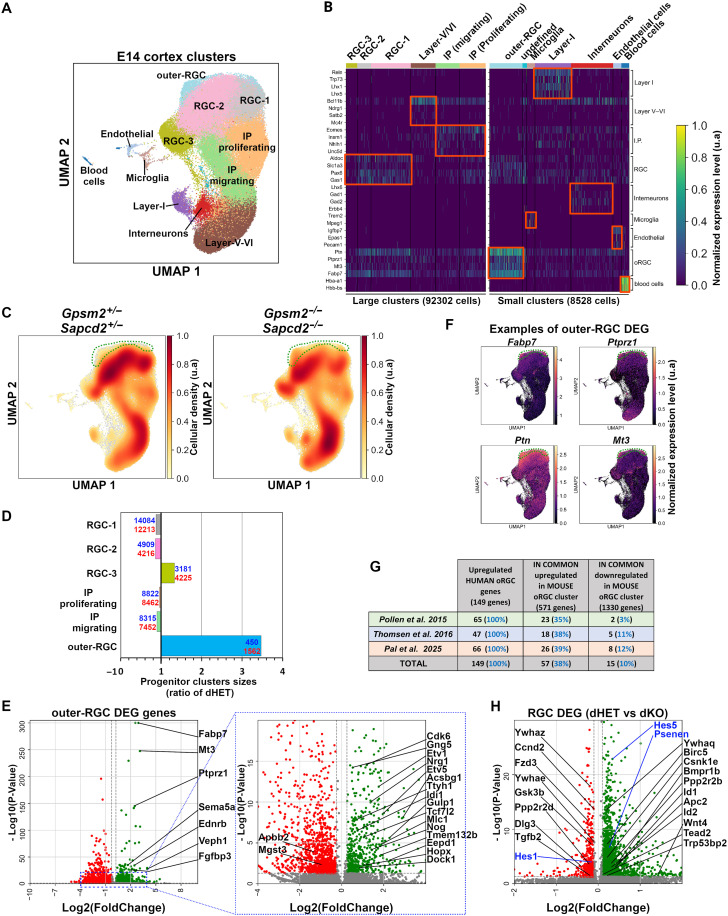
scRNA-seq reveals overproduction of oRGCs and Hippo pathway dysregulation. (**A**) UMAP of E14.5 cortex scRNA-seq clusters identified by Leiden algorithm ran over all cells pooled regardless of their genotype (dHET or dKO). (**B**) Heatmap of representative cell markers used to identify cell types corresponding to each Leiden cluster. To help visualize the heatmap, the smallest clusters were separated into two groups (large and small clusters). (**C**) UMAP of dHET (left) and dKO (right) showing the density of cells over all clusters in each genotype. The dashed green line circles the oRGC clusters, which present a higher density in dKO condition. (**D**) Bar graph of progenitor cluster size ratios between dHET and dKO. Absolute numbers of cells for each cluster are depicted in blue for dHET and red for dKO genotype. (**E**) Volcano plot showing the DEG in the oRGC cluster. Genes in common between human oRGC identified in Pollen *et al.* and the mouse oRGC clusters are identified. *Fabp7* and *Ptprz1* human oRGC markers are among the top 10 genes up-regulated in oRGC clusters. (**F**) Examples of oRGC DEG level of expression mapped on the general UMAP. In addition to human oRGC markers *Ptprz1* and *Fabp7*, the ligand of *Ptprz1* receptor Ptn is the second top DEG found in oRGC clusters. (**G**) Table of differentially expressed genes (DEG) found in oRGC clusters compared to the list of human oRGC-specific genes found in Pollen *et al.* (*41*) Thomsen *et al.* (*42*), and Pal *et al.* (*43*). (**H**) Volcano plot indicating the list of DEG between dHET and dKO RGC clusters. Black and blue genes are associated with the Hippo and Notch pathway, respectively.

Given the drastic increase in vertical divisions observed in the embryonic cortex after inactivation of *Gpsm2* and *Sapcd2* without a change in tissue polarity, dKO embryos offer a unique opportunity to explore the specific impact of division orientation on the generation of different types of basal progenitors (IP versus oRGCs). To do this, we conducted scRNA-seq analyses on dissociated E14.5 cortices and compared the transcriptional signature of cell populations between dHET and dKO littermates. We first performed an unbiased clustering of all cells, regardless of the genotypes, resulting in 13 distinct cell clusters (Fig. 4, A and B). Subsequently, we stratified these clusters by genotype to evaluate cell density variations between dHET and dKO samples. Notably, we observed a substantial 3.4-fold increase in cell density within a cluster situated nearby RGC clusters in dKO compared to dHET samples (Fig. 4, C and D). Given our suspicion that this population might correspond to the overproduced basal progenitors in the dKO cortex, we examined known markers associated with IPs or oRGCs. This cluster did not exhibit expression of the IP marker *Tbr2/Eomes* (fig. S9), but *Hopx,* the main oRGC marker in mice, was significantly up-regulated in the dKO sample (Log2FC = 1.0, adj. *P* value = 0.004) (Fig. 4E). While extensive scRNA-seq characterization of oRGCs has been conducted in human embryonic cortical cells, much less is known about this progenitor subtype in rodents, where they are normally sparse (*41*, *42*). Thus, we cross-referenced differentially expressed genes (DEGs) with the list of genes identified as human oRGC specific genes by Pollen *et al.* (*41*) and Thomsen *et al.* (*42*). Despite the evolutionary distance between human and mouse, we found that 23 of 65 genes (35%) and 18 of 47 genes (38%) are up-regulated in the cluster identified in the dKO mouse cortex, therefore referred to as oRGC cluster (Fig. 4, E and G, and table S2). Among these, the mouse oRGC markers *Hopx, Ptprz1* and *Fabp7* were up-regulated (Fig. 4E). In addition, we noted up-regulation of the *Ptprz1* receptor ligand *Ptn* (Log2FC = 2.56 and adj. *P* value = 1.00 × 10^−300^) and the metal homeostasis protein metallothionein-3 in the oRGC cluster (Fig. 4F and table S2). In a recent study, a list of oRGC-specific genes targeted by human accelerated regions and human gain enhancers was identified by Pal *et al.* (*43*), providing another gene set potentially involved in oRGC generation and cortical expansion. Among this list of 66 genes reported in humans, 39% (26 of 66) of them are also up-regulated in the dKO mouse oRGC cluster (Fig. 4G), including the zinc-finger transcription factor *Prdm16* (Log2FC = 1.62 and adj. *P* value = 3.57 × 10^−18^) involved in fetal and postnatal neural stem cell maintenance and laminar specification of upper layer cortical neurons (*44*, *45*). To validate these results, we performed in situ hybridization for *Ptprz1* and *Fabp7,* two of the strongest hits identified by scRNA-seq. We found that the signal for both is expanded and more intense in the developing neocortex of dKOs, with the strongest positive cells located basally (fig. S10, A and B red brackets). However, in contrast to humans, we found that *Ptprz1* and *Fabp7* are not restricted to oRGC in the developing mouse neocortex but are also expressed in apical progenitors located in the ventricular zone (fig. S10B, left).

To determine whether the *Ptprz1* expressing cells are actively proliferating, we injected EdU into P0 animals and coimmunostained for PTPRZ1. We found that the number of EdU^+^/PTPRZ1^+^ cells was not significantly changed in the VZ/SVZ, but there was a more than threefold increase in double-positive cells outside the VZ/SVZ in dKO embryos compared to dHET littermates (fig. S10, C to E). Next, we compared the number of EdU^+^ cells coexpressing either SOX2*,* a RGC marker, or TBR2*,* an IP marker. We found a significant increase in the proportion and density of SOX2^+^/EdU^+^ cells in dKO (fig. S11, A to C), whereas the proportion of TBR2^+^/EdU^+^ cells is not significantly affected, although their density is increased (fig. S11, D to F). Thus, even though the density of all progenitor cells (RGC + IP) is increased in dKO cortex, only oRGCs are proportionally increased (fig. S10E).

### Altering division orientation dysregulates pathways controlling neural progenitor proliferation

We next sought to identify signaling pathways that may participate in the overproduction of oRGCs in the dKO neocortex. As apical RGCs give rise to oRGCs, we examined DEGs in the global RGC clusters between dHET and dKO. These DEGs were cross-referenced with known genes associated with signaling pathways documented in the Kyoto Encyclopedia of Genes and Genomes. Consistent with previous reports showing that activation of YAP1 and WW domain containing transcription regulator 1 (also known as TAZ) promotes production of basal progenitors (*7*) and subcortical heterotopia with hemorrhagic hydrocephalus (*46*), as found in the *Sapcd2/Gpsm2* dKO (fig. S6A), we observed differential expression of 24 of 137 Hippo pathway genes (17.5%) in dKO RGCs compared to dHET (Fig. 4G). In addition, we found differential expression of 12 of 63 Notch pathway genes (19%), consistent with previous work showing asymmetric activation of the Notch pathway in vertical divisions (*21*, *47*–*49*).

**Fig. 5.**
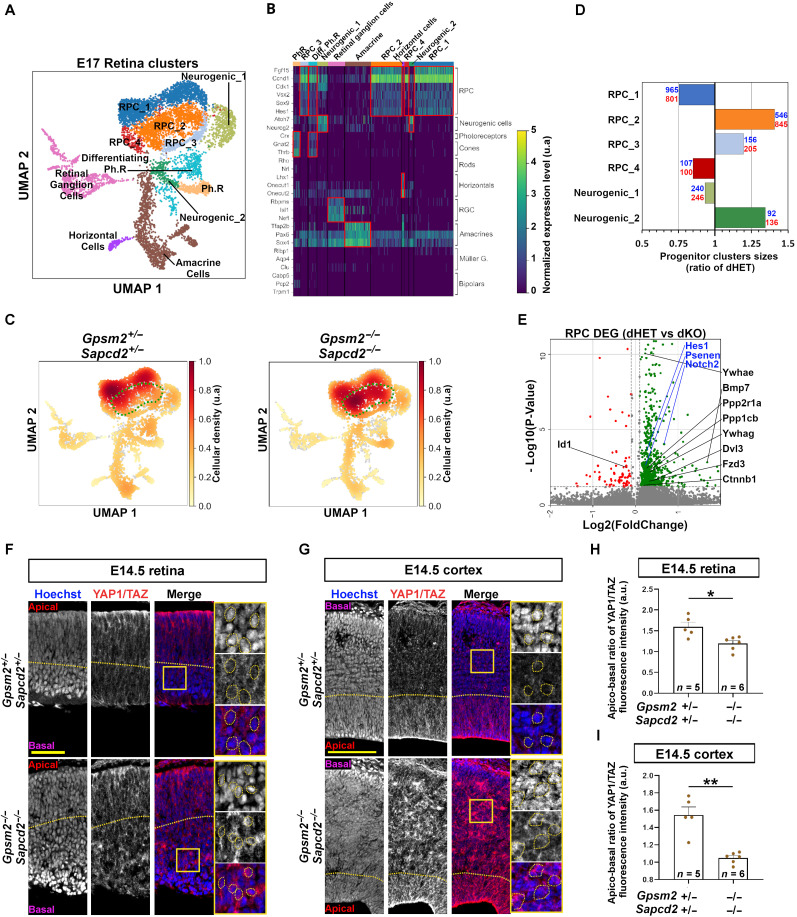
scRNA-seq in dHET and dKO retinas reveals Notch and Hippo pathway dysregulation. (**A**) UMAP of E17.5 retina scRNA-seq clusters identified by Leiden algorithm ran over all cells pooled regardless of their genotype (dHET or dKO). (**B**) Heatmap of representative cell markers used to identify cell types corresponding to each Leiden cluster. (**C**) UMAP of dHET (left) and dKO (right) showing the density repartition over all clusters in each genotype. The dashed green line circles the portion of the retinal progenitor cell (RPC) cluster that presents a stronger density of cells in dKO condition. (**D**) Bar graph of progenitor cluster size ratios between dHET and dKO. Absolute numbers of cells for each cluster are depicted in blue for dHET and red for dKO genotype. (**E**) Volcano plot indicating the list of DEG between dHET and dKO RPCs. Black and blue genes are associated with the Hippo and Notch pathway respectively. (**F** and **G**) Immunolabeling for YAP1/TAZ in developing retina (E) and neocortex (F). Scale bars, 50 (E) and 100 μm (F). (**H** and **I**) Quantification of YAP1/TAZ mean fluorescence intensity ratio between apical and basal regions of developing retina (G) and neocortex (H). Comparisons between groups were done using non-parametric Mann-Whitney test (*P < 0.05 and **P < 0.01).

We next carried out scRNA-seq on E17.5 dKO and dHET littermate retinas, a time when basal divisions are detected in dKO. We defined 12 clusters corresponding to the different cell types produced at this developmental stage (Fig. 5, A and B) and then compared cell density between dHET and dKO cell populations. As observed in the neocortex, we found a shift in dKO retinal progenitor cell density in the RPC_2 and RPC_3 clusters, suggesting a change in their molecular signature (Fig. 5, C and D). Consistently, DEG analysis between dHET and dKO retinal progenitors revealed up-regulation of several Hippo and Notch pathway components, including *Ywhae*, *Ywhag*, *Dvl3*, *Notch2*, *Psenen*, and *Hes1* (Fig. 5E), similar to the changes observed in the dKO cortex. Our previous study demonstrated that SAPCD2 can interact with Hippo pathway regulators such as AMOT in the retina (*24*), providing an additional link between spindle orientation regulator and the Hippo pathway.

The Hippo pathway is a master regulator of tissue size (*50*–*53*). One consequence of Hippo pathway inactivation is the release of inhibition of its final effectors YAP1 and TAZ, which translocate to the nucleus and promote proliferation. Thus, we compared expression of YAP1 and TAZ in dHET and dKO developing neocortex and retina. Using an antibody recognizing both YAP1 and TAZ, we found a strong increase in their expression level in the basal regions of the E14.5 retina and neocortex, including an increase in nuclear signal (Fig. 5, F to I). Together, these results suggest that loss of *Sapcd2* and *Gpsm2* correlates with the release of inhibition of the Hippo pathway effectors and activation of the Notch pathway in both the retina and neocortex, driving hyperplasia in these tissues.

### YAP1 induces ectopic basal progenitors and over proliferation in the developing mouse retina

**Fig. 6.**
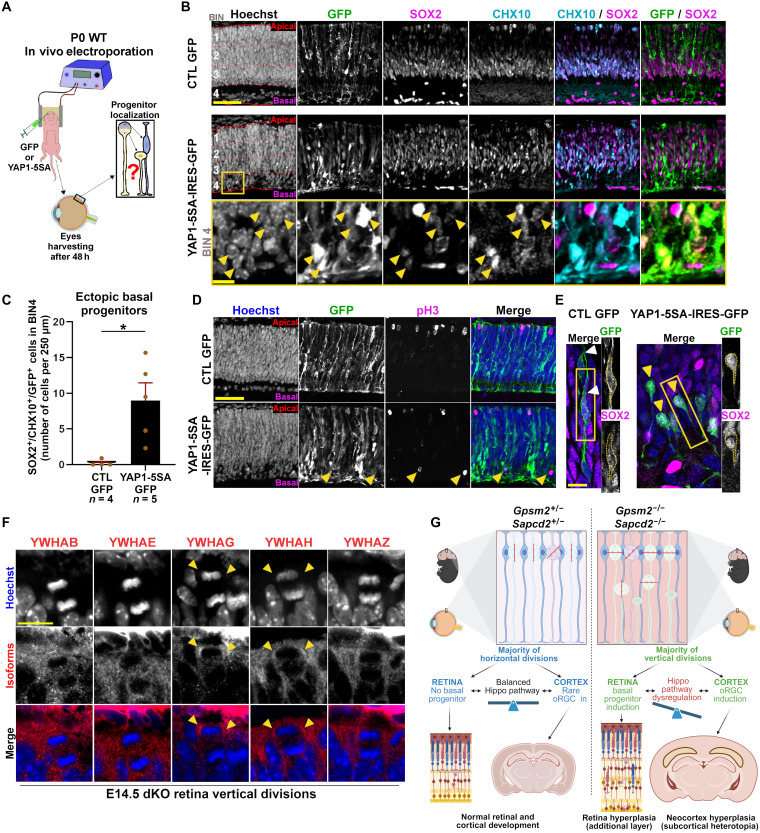
Constitutively active YAP1 is sufficient to generate basal progenitors in developing mouse retina. (**A**) Illustration of experimental procedure to test the effect of YAP1 gain of function on RPC localization. (**B**) Coimmunolabeling of the progenitor markers SOX2 and CHX10 48 hours after P0 retinal electroporation of either pCAG-IRES-GFP or pCAG-YAP1-5SA-IRES-GFP plasmids. Note the presence of SOX2^+^/CHX10^+^ double-positive cells in the most basal quarter (BIN4) of the developing retina upon YAP1-5SA overexpression (yellow arrowheads), while none of them are found in GFP controls. Scale bar, 10 μm. (**C**) Quantification of the number of GFP^+^/SOX2^+^/CHX10^+^ triple-positive cells in BIN4 48 hours after electroporation of either pCAG-IRES-GFP or pCAG-YAP1-5SA-IRES-GFP. Comparisons between groups were done using nonparametric Mann-Whitney test (*P < 0.05). (**D**) Phospho–histone-3 (pH3) immunolabeling on retinal sections showing ectopic basally dividing progenitors upon YAP1-5SA overexpression. Scale bar, 50 μm. (**E**) High-resolution images of GFP staining showing the loss of apical process in YAP1-5SA–expressing progenitors. White arrowheads point on apical processes in pCAG-IRES-GFP electroporated bipolar progenitors, yellow arrowheads point on unipolar progenitors with a basal process only in retinas electroporated with pCAG-YAP1-5SA-IRES-GFP. Expression of SOX2 is used to identify progenitors of electroporated cells. Scale bar, 10 μm. (**F**) Immunolabeling for the different isoforms (YWHA-B, YWHA-E, YWHA-G, YWHA-H, and YWHA-Z) of the Hippo pathway regulator YWHA on E14.5 retinas showing apical accumulation (arrowheads) of the YWHAG and YWHAH isoforms in vertical divisions. (**G**) A graphical summary of dKO phenotypes. Created in BioRender. Cayouette, M. (2026) https://BioRender.com/8njqxsy.

Beyond its role in tissue size regulation, the Hippo pathway is necessary and sufficient to produce basal progenitors in the developing neocortex (*7*). As we find an increase in basal progenitor generation and YAP1/TAZ activation in both the neocortex and retina of dKO mice, we next wondered whether basal progenitors could be ectopically generated in the developing retina by manipulating the Hippo pathway. We electroporated P0 retinas with an expression vector coding for a constitutively active form of YAP1 [YAP1-5SA (*54*)] (Fig. 6A). Two days after electroporation, we stained retinal sections for the progenitor markers CHX10 and SOX2 and compared YAP1-5SA electroporated retinas with green fluorescent protein (GFP) controls. We found that inhibition of the Hippo pathway through overexpression of YAP1-5SA led to progenitor cells localizing in the basal quarter of the retina, which was never observed in GFP controls (Fig. 6, B and C). In addition, we found pH3^+^ cells on the basal side of the retina in YAP1-5SA condition (Fig. 6D), indicating ectopic mitosis as we observed in E14.5 dKO retinas (Fig. 2B). These ectopic basal progenitors observed after YAP1-5SA expression lacked an apical end-foot (Fig. 6E), like retinal progenitors in dKO (fig. S5) and oRGCs in the cortex (*55*). Next, we wondered if YAP-5SA–induced basal progenitors go on to overproduce retinal cells. To address this question, we electroporated P0 WT retina in vivo with YAP1-5SA and waited until P21 to analyze the identity of electroporated cells (GFP^+^). Reminiscent of what we observed in adult dKO retinas, YAP1-5SA overexpression triggered a massive overproduction of mis-laminated cells composed of PAX6-positive amacrine cells (fig. S12A) and CHX10-positive bipolar cells (fig. S12A). However, unlike in dKO retinas, we noticed overproduction of LHX2-positive Müller glial cells (fig. S12B), phenocopying the effect of YAP1-5SA overexpression in the cortex where it induces astrocytes (*56*). As previously reported with overexpression of this construct in the developing neocortex, we noticed a non–cell autonomous effect of YAP1-5SA overexpression in the developing retina, with many overproduced cells not expressing the GFP reporter (*56*).

### Asymmetric inheritance of Hippo pathway regulators in vertical divisions

We next asked whether reorientation of the mitotic spindle might lead to asymmetric segregation of Hippo regulators in one of the daughter cells of vertical divisions. To explore this possibility, we took advantage of our IP-MS dataset to identify candidate Hippo regulators associated with PAR3 at the apical domain of the developing retina and cortex (table S1). In this list, we found several isoforms of 14-3-3 protein (also known as YWHA or PAR5) that coimmunoprecipitated with PAR3 in the P0 retina and cortex. Notably, we found that two isoforms of these candidates (YWHAG and YWHAH) localize at the apical surface of dividing progenitors in the developing cortex and retina, consequently becoming asymmetrically segregated in the apical daughter cell of vertical divisions (Fig. 6F). These results suggest that reorientation of the mitotic spindle may generate asymmetric Hippo signaling between daughter cells, leading to translocation of YAP/TAZ in the nucleus and Hippo pathway inhibition in the basal cell, thereby enhancing its proliferative potential (Fig. 6G).

### Cell division orientation regulates the production of basal progenitors and retinal tissue expansion in humans and nonhuman primates

Given that *Gpsm2/Sapcd2* inactivation promotes oRGC production in mice, we wondered if the increased oRGC numbers in humans could be driven by a down-regulation of these mitotic spindle regulators in RGCs. To address this question, we first reanalyzed a published dataset comparing expression of 15,576 homologous genes between human and mouse neocortical development, and their enrichment in RGCs compared to other brain cell types (*57*). As proof of concept, this study reports increased expression of *Hopx* and *Ptprz1* in humans compared to mice, consistent with an increased number of oRGCs (fig. S13A). We found that 75% of the known regulators of mitotic spindle orientation enriched in RGCs are down-regulated in humans compared to mice (fig. S13A). Among those, we found *PAR3*, *INSC*, *ASPM*, as well as *SAPCD2* and the *GPSM2* paralog *GPSM1* (*AGS3*) (Fig. 4H). Second, we reanalyzed a published scRNA-seq atlas of human cortical organoids at different time points of development (*58*). We found that human apical RGCs down-regulate *SAPCD2* and *GPSM2* after 2 months in culture (fig. S13B), which correspond to the time point of oRGC emergence in these organoids. These results suggest that down-regulation of *SAPCD2* and *GPSM1/GPSM2* in human RGCs may have led to an evolutionary increase in the frequency of vertical divisions and consequent overproduction of oRGCs to drive brain size expansion.

**Fig. 7.**
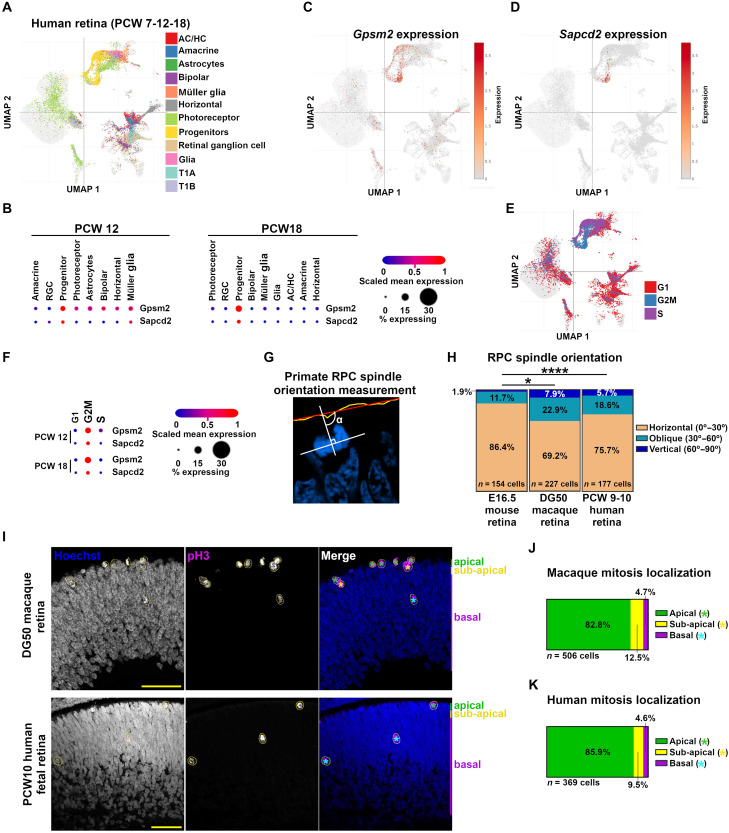
Increased proportions of reoriented progenitor divisions, correlating with the presence of basal progenitors in the rhesus macaque and human retinas. (**A**) UMAP of Human retina cell type clusters identified in all cells combined from PCW7, PCW12, and PCW18 time points from Soucy *et al.* (*59*) using the Broad Institute’s single-cell portal. (**B**) *Gpsm2* and *Sapcd2* expression levels by cell types showing enrichment in progenitors. (**C** and **D**) UMAP illustrating *Gpsm2* (C) and *Sapcd2* (D) expression levels. (**E**) UMAP of cell cycle states enrichment by cell types. (**F**) *Gpsm2* and *Sapcd2* expression levels as a function of cell cycle phases. (**G**) Illustration of mitotic spindle orientation measurement in human and nonhuman primates based on chromosome alignment to the mitotic plane. (**H**) Retinal progenitor mitotic spindle orientation comparison between aged-matched mouse E16.5, DG50, macaque and PCW9-10 human developing retinas. (**I**) Immunolabeling for phosphorylated histone 3 (pH3) in developing macaque (upper panel) and human (lower panel) retinas. Scale bars, 50 μm. (**J** and **K**) Quantification of pH3^+^ RPC localization relative to the distance from the apical border in nonhuman primates (J) and humans (K). Apical progenitors are in contact with the apical membrane, subapical progenitors are separated from the apical membrane by one nucleus, whereas basal progenitors are more than one cell away from the apical membrane in the progenitor layer. Comparisons between groups were done using a one-way ANOVA test followed by Dunn’s correction for multiple comparisons. (*P < 0.05 and ****P < 0.0001).

If the above model is correct, we predicted that animals with larger retinas would have a higher proportion of vertical divisions and display basal progenitors, which have not been reported in the mammalian retina, but arise in *Gpsm2/Sapcd2* dKO. First, we analyzed published single-cell RNA-seq data from the developing human retina (*59*) and found that *Gpsm2* and *Sapcd2* transcripts are enriched in retinal progenitors (Fig. 7, B to D), particularly during the G_2_/M phase (Fig. 7, E and F), consistent with their roles in mitotic spindle orientation. Notably, however, the proportion of progenitors expressing *Gpsm2* and *Sapcd2* was lower in the human dataset at PCW12 (6.7 and 1.8%, respectively; Fig. 7B) compared with the mouse dataset (*60*), where expression was observed in 34.4 and 30.5% of progenitors, respectively. Next, we analyzed retinal progenitor cell division orientation and location in the developing rhesus macaque (*Macaca mulatta*) and human retinas. We found that the macaque retina at 50 days gestational age (DG50) and the human retina at postconceptional weeks (PCW) 9 to 10 present three to four times more vertical and about two times more oblique divisions than the age-matched E16.5 mouse retina (Fig. 7, G and H). Consistent with this increase in reoriented divisions, we found pH3^+^ mitotic progenitor cells on the basal part of the macaque (4.7%) and human (4.6%) retinas (Fig. 7I), and ~10% of subapical divisions undergoing mitosis at least one cell body away from the apical surface (Fig. 7, I to K). These results indicate that the number of vertical divisions and the emergence of basal progenitors correlates with increased retinal size in both humans and nonhuman primates.

## DISCUSSION

In this study, we demonstrate that GPSM2 and SAPCD2 are critical components of a regulatory network of neural progenitor cell division orientation. While the loss of each gene individually causes mild disruptions in division orientation and cell fate, we find that simultaneous inactivation of *Sapcd2* and *Gpsm2* drastically shifts mitotic spindle orientation of neural progenitors from predominantly horizontal to mostly vertical in both the developing retina and cortex. This shift leads to an increased number of cortical and retinal progenitors, including an overproduction of basally dividing progenitors. In the developing neocortex, these basal progenitors express markers associated with oRGCs in humans. Consequently, *Sapcd2/Gpsm2* dKO mice exhibit an enlarged adult neocortex and retina, including subcortical heterotopia and additional nuclear and plexiform layers in the retina. Mechanistically, our data suggest that asymmetric inheritance of Hippo regulators during vertical divisions may lead to translocation of YAP/TAZ in the nucleus and inhibition of the Hippo pathway in the basal cell, which delaminates and continues to proliferate to expand neuronal production. Last, we show that the developing human and nonhuman primate retinas contain more reoriented divisions than mice and display basally dividing progenitors, which are not normally found in the mouse retina. Together, these results suggest a model in which GPSM2 and SAPCD2 cooperate to maintain the mitotic spindle parallel to the plane of the neuroepithelium, thereby regulating tissue size.

Previous studies have shown that GPSM2, through its TPR and GoLoco domains, anchors the NuMA-dynein complex to the cell cortex, thereby generating pulling forces on aster microtubules to align the mitotic spindle (*61*, *62*). SAPCD2, in turn, modulates this process by preventing GPSM2 localization at the apical cortex, a mechanism previously shown to bias divisions toward the planar axis in retinal progenitors (*24*). Our data reveal that when both proteins are inactivated, cortical anchoring of aster microtubules is likely destabilized, resulting in spindle tumbling along the apico-basal axis. To reconcile our findings with previous reports suggesting antagonism between GPSM2 and SAPCD2, we propose that these proteins function in a broader balanced regulatory network, where the inhibitory role of SAPCD2 ensures GPSM2 is restricted to cortical sites permissive for planar spindle alignment. In this view, rather than acting in opposition, the two proteins cooperate to stabilize spindle orientation horizontally within the neuroepithelium. In addition, we cannot exclude that other pathways contribute to control spindle orientation and that SAPCD2 regulates their activity. For example, future studies on the potential role of GPSM1-SAPCD2 interactions (*24*) could help clarify this possibility.

### The role of cell morphology in the regulation of neural progenitor division orientation

Cell shape has long been thought to regulate mitotic spindle orientation (*63*–*65*). This principle, first established by Hertwig a century ago, posits that cells undergo mitosis with their mitotic spindle aligned along their long axis. While numerous studies have further documented this phenomenon in various species and in micropattern models in vitro [for review, see (*63*)], its relevance to CNS development remains controversial. Neural progenitors predominantly divide with their spindle perpendicular to their long apicobasal axis and disruption of regulators of division orientation generally leads to only modest increase in mitotic spindles aligned parallel to this long axis (*25*, *35*, *66*–*68*). These observations have led to the general idea that horizontal is the default spindle orientation in neuroepithelial cells, which is contrary to the Hertwig principle. However, our study now demonstrates that GPSM2/SAPCD2 function to actively maintain the spindle horizontal and that their inactivation triggers reorientation of most progenitor divisions along the vertical (apicobasal) axis, suggesting that this is the default orientation, in agreement with Hertwig’s model. In other studies where regulators of cell division orientation were manipulated, the distribution of division angles was at best randomized. In contrast, we find here that the distribution of division orientations is inverted in dKO compared to controls, further suggesting that vertical is the default orientation. In this model, negative regulators of GPSM2 and SAPCD2 would release the tension keeping the mitotic spindle horizontal, triggering a reorientation along oblique and vertical axes and the production of proliferative asymmetric cell divisions. Future studies exploring negative regulators of SAPCD2 and GPSM2 may shed light on the mechanisms involved in balancing symmetric and asymmetric cell divisions in diverse developmental contexts and animal species.

### Generation of basal progenitors by vertical divisions and impact of the Hippo pathway

The *Gpsm2/Sapcd2* dKO mouse model is characterized by the predominance of vertical divisions, providing a convenient system to study the role of these normally rare divisions. Our data now provides compelling evidence that vertical divisions preferentially promote the generation of oRGCs during corticogenesis. Given the important role of oRGCs in the enlargement of the neocortex in primates (*69*, *70*), these results suggest that the scarcity of vertical divisions in the developing mouse cortex explains the low number of basal divisions and limited cortical expansion. Consistently, the level of expression of many known regulators of mitotic spindle orientation, including *Sapcd2* and *Gpsm1*, is lower in the developing human brain compared to mice (*57*). We propose that this may have led to an increased frequency of vertical divisions and the emergence of oRGCs in the developing human cortex. This model is consistent with a study reporting that reorientated (vertical spindle) RGC divisions are more frequent in the human cortex than rodents, which drives overproduction of oRGCs that fuel cortical expansion in humans (*9*). In addition, the increased length of human corticogenesis compared to rodent corticogenesis provides an extended window for human vertical divisions to generate oRGCs. Our findings that the macaque and human retinas contain more reoriented divisions than the mouse, and feature the emergence of basally dividing cells, which are not normally found in the mouse retina, is consistent with this model and highlight similarities between cortical and retinal neurogenesis that were previously unrecognized.

Recent investigations have highlighted the crucial role of the Hippo pathway in basal progenitor production in developing neocortex (*7*). In addition, reducing the population of cortical basal progenitors by inactivating YAP1/TAZ results in the generation of fewer upper layer neurons (*7*, *71*). In our study, we conversely demonstrate that an increase in vertical divisions in *Gpsm2/Sapcd2* dKO mice leads to YAP1/TAZ over-activation in the retina and cortex (Figs. 4 and 6), suggesting a role in tissue expansion. Consistently, we report that expression of YAP1-5SA in retinal progenitors is sufficient to induce basal divisions and stimulate overproduction of neurons. While our data show that the Hippo regulators YWHAG and YWHAH are asymmetrically inherited by the apical daughter cell of vertical divisions, suggesting a model in which YAP/TAZ are free to translocate to the nucleus of the basal cell to drive proliferation, future experiments will be required to directly test this idea.

Why overproduction of basal progenitors leads to heterotopia will also require further investigations. In the cortex, we speculate that the increased production of basal progenitors progressively depletes the RGC pool, thereby reducing the scaffold and guidance signals required for proper neuronal migration. As a result, many late-born neurons might fail to reach their appropriate laminar position and instead accumulate near the ventricular zone, ultimately forming subcortical heterotopia. In the mouse retina, the mechanisms of neuronal migration and lamination are less well characterized, but we propose that neurons generated ectopically on the basal side of the tissue lack access to the spatial and molecular cues normally provided by the apical environment, impairing their ability to integrate into the correct layers. Additional experiments will be necessary to test these hypotheses.

Our results are consistent with studies showing that conditional deletion of the polarity protein PAR3 increases basal progenitors, leading to cortex enlargement and Hippo pathway dysregulation (*46*). Although brain-specific conditional inactivation of PAR3 increases the proportion of vertical divisions, it remains unclear whether the observed phenotypes are caused by changes in division orientation, a loss of polarity, or both. In the *Gpsm2/Sapcd2* dKO, we find that some cells located on the basal side of the neuroepithelium have lost their apical process and display a unipolar morphology. These results support a model in which reoriented divisions give rise to daughter cells that detach from the apical surface and reach basal parts of the neuroepithelium where they contribute to tissue expansion in both the retina and cortex. We show that neuroepithelial polarity is unaffected in dKO, pointing more directly to a role for division orientation per se in basal progenitor generation and tissue size regulation.

Mouse mutants of the *echinoderm microtubule-associated protein like 1* (*Eml1*) gene were found to contain subcortical heterotopia, and also exhibit retinal defects characterized by basally located progenitors, increased retinal thickness, and photoreceptor delamination, reminiscent of the phenotypes observed in *Gpsm2/Sapcd2* dKO mice (*72*). Although it remains unknown whether *Eml1* mutants show more vertical divisions in the retina, it is interesting to note that heterozygous mutations in *Eml1* have been associated with an increase in vertical divisions, Hippo pathway inhibition, and oRGC production in human cortical organoids (*38*). On the other hand, *Eml1* mutant retinas undergo increased neurodegeneration, leading to the normalization of retina thickness 1 month after birth (*72*). This normalization does not occur in *Gpsm2/Sapcd2* dKO retinas, suggesting that *Eml1* may have additional functions beyond regulation of division orientation. In conclusion, our findings establish division orientation as a conserved driver of neural expansion via basal progenitor regulation, paving the way to identify how spindle orientation pathways scale neural tissue size across evolution.

## MATERIALS AND METHODS

### Animals

#### Mice

All animal work was performed in accordance with guidelines from the Institut de Recherches Cliniques de Montréal animal care committee (no. 2022-1154) and the Canadian Council on Animal Care. *Gpsm2/Sapcd2* double mutants were generated by crossing Gpsm2^+/−^ (*73*) with *Sapcd2*^−/−^ (*74*) followed by inbreeding of *Gpsm2*^+/−^*; Sapcd2*^+/−^ littermates. *Gpsm2/Sapcd2* double mutants of either sex were used in this study.

#### Macaques

All animal procedures adhered to the requirements of the Animal Welfare Act and were approved in advance by the Institutional Animal Care and Use Committee (IACUC) at the University of California, Davis (no. A-3433-01). Healthy adult female rhesus monkeys (*M. mulatta*) were bred and confirmed pregnant using established protocols (*75*). Normal embryonic and fetal development was verified throughout gestation via ultrasound until the time of tissue collection (*75*). Dams were scheduled for hysterotomy at 50 days gestational age. Following surgery, dams were returned to the breeding colony. Retinal samples were then fixed overnight at 4°C in modified Carnoy’s fixative (ethanol, formaldehyde, and acetic acid), followed by dehydration through graded ethanol solutions, clearing with xylene, and embedding in paraffin blocks. Retinas were sectioned (10 μm) in the horizontal/transverse plane. Two animals and sections of both retinas were used in this study.

#### Humans

Human embryo and fetal samples used in this study were obtained from terminations of pregnancy with written and informed consent from all sample donors. Samples were provided by INSERM’s HuDeCA Biobank and used in compliance with French regulations. Authorization to use these tissues was granted by the French agency for biomedical research (Agence de la Biomédecine, Saint-Denis La Plaine, France; N° PFS19-012) and the INSERM Ethics Committee (IRB00003888).

The slides of human retina were thawed in a humid chamber for approximately 10 min. To remove the OCT, they were washed with 1× phosphate-buffered saline (PBS; three washes of 10 min each). Permeabilization and blocking were carried out for 1 hour using PBS-gelatin 0.2% Triton-X 100 0.5% (PBSGT 0.5%). Sections were next incubated overnight at room temperature and in a humid chamber with primary antibody (H3P, 1:1000, Cell Signaling no. 9701) diluted in PBSGT 0.5%. Afterward, the slides were washed again with PBSGT 0.5% (three washes of 10 min each), and incubated for 2 hours at room temperature, protected from light with a secondary antibody [donkey anti-rabbit immunoglobulin G (IgG) H&L (Alexa Fluor 555), 1:500, Abcam no. ab150062]. The secondary antibody was subsequently washed with PBST 0.5%. The slides were then mounted with a coverslip using Mowiol mounting media. Image stacks were acquired using an Olympus FV3000 confocal microscope. Five fetuses and sections of both retinas were used in this study.

### Mitotic spindle orientation measurements

Mitotic spindle orientations were measured as previously published (*22*, *24*). In brief, centrosomes were stained with anti-NuMA (Novus Biologicals, NB500-174SS) antibody and DNA labeled with Hoechst. The orientation of the spindle axis was measured using a MATLAB program (The Mathworks Inc., Natick, MA) by calculating the *x*, *y*, and *z* coordinates of the centrosome pairs relative to the plane of the neuroepithelium corresponding to the interface between the retinal pigmented epithelium and the neural retina or between lateral ventricle and neocortex. We analyzed the centrosome pairs in cells in either metaphase or anaphase-telophase.

### In situ hybridization

Digoxigenin-labeled RNA probes were synthesized from E14.5 brain cDNA preparations by polymerase chain reaction (PCR) amplification using LongAmp PCR kit (NEB, E5200S) and the following primers (Table 1). Probes templates were then cloned into pCRII-TOPO backbone using TOPO TA cloning kit (Thermo Fisher Scientific, K461020). The plasmid was linearized with BamH I to prepare the template DNA for the probe synthesis. Then sense and antisense probes were produced using T7 and SP6 RNA polymerase and purified on Sephadex G-50 RNA purification columns (Roche, 11274015001). Embryonic E14.5 and postnatal P0 heads were fixed in 4% paraformaldehyde (PFA) overnight, cryopreserved in PBS 20% sucrose, frozen in OCT and sectioned at 12 μm thickness. Sections were incubated overnight with hybridization buffer {50% (v/v) formamide, 10% (w/v) dextran sulfate, ribosomal RNA (rRNA; 1 mg/ml), 1× Denhardt’s solution, and 1× salt solution [200 mM NaCl, 10 mM tris (pH 7.5), 5 mM sodium phosphate buffer, and 5 mM EDTA]} at 65°C with RNA probe (100 ng/ml). The probes were detected with an alkaline phosphatase–conjugated anti-digoxigenin antibody (1:2500, Roche). The AP activity was revealed using 4-nitro blue tetrazolium chloride/5-bromo-4-chloro-3-indolyl-phosphate substrate solution (Roche).

**Table 1.** Sequences of PRC primers used for In situ hybridization probes production.

Probe	Forward	Reverse
pCRII-TOPO-FABP7	gtagatgctttctgcgcaac	cataacagcgaacagcaacg
pCRII-TOPO-PTPRZ1	ctccgggatctccacggtcc	gcctgggcagcctttctcac

### Histology and immunofluorescence

Mouse eyes and brains were enucleated in PBS and fixed by immersion in freshly prepared 4% PFA in PBS. Eyes were cryoprotected overnight at 4°C in sucrose 20%, embedded in OCT, and stored at −80°C until sectioning. Mouse brains were fixed in 4% PFA in phosphate buffer at 4°C for 2 to 4 hours followed by overnight cryoprotection in 20% sucrose and embedded in OCT. Eyes and brains were cryosectioned at 20 μm and frozen back at −80°C until immunostaining. Slides were preincubated for 30 min in blocking buffer [2% bovine serum albumin (BSA) and 0.1% Triton-X 100] and incubated overnight at room temperature with primary antibody (listed in Table 2) diluted in blocking buffer. Primary antibodies were detected using appropriate secondary antibodies conjugated with Alexa Fluor 488, 555, or 594 (1:1000; Invitrogen) diluted in PBS at room temperature for 1 hour. In all cases, nuclei were stained with Hoechst 33342 (1:20,000, Molecular Probes). The slides were mounted in Mowiol.

**Table 2.** List of antibodies used for immunofluorescence labeling.

Antigen	Species	Dilution	Company	Cat. number
BRN2/POU3F2	Rabbit	1/500	Cell Signaling Technology	NEB#12137S
BrdU	Mouse	1/250	EXBIO	11-286-C100
BRN3a	Guinea pig	1/500	Synaptic Systems	411004
CHX10	Sheep	1/500	Exalpha Biologicals	X1180P
Cleaved caspase 3	Rabbit	1/100	Cell Signaling Technology	NEB#9661
Cone arrestin	Rabbit	1/1000	LsBio	LS-B9272
CTIP2	Rat	1/400	Abcam Biochemicals	ab18465
LHX2	Rabbit	1/200	Thermo Fisher Scientific	PA5-78287
LIM1	Rabbit	1/500	Abcam	ab229474
NRL	Goat	1/500	R&D Systems	AF2945
NuMA	Rabbit	1/200	Novus Biologicals	NB500-174SS
OLIG2	Rabbit	1/500	PhosphoSolutions	AB_2492193
PAX6	Rabbit	1/200	Millipore Sigma	AB2237
PAR3	Rabbit	1/200	EMD Millipore	07-330
Parvalbumin	Rabbit	1/1000	Swant	PV27a
PODXL	Goat	1/500	R&D Systems	AF1556
pH3	Rabbit	1/400	EMD Millipore	06-570
PTPRZ1	Rabbit	1/500	Millipore Sigma	HPA015103
SOX2	Rabbit	1/200	Abcam Biochemicals	AB97959
SOX9	Rabbit	1/500	Millipore Sigma	AB5535
TBR2	Rabbit	1/250	Abcam	ab183991
ZO-1	Rat	1/200	DSHB	R26-4C-s
YAP1 and TAZ	Rabbit	1/500	Cell Signaling	D24E4
Rod arrestin (SAG)	Mouse	1/100	Santa Cruz	sc-166383
GAD67	Mouse	1/200	Millipore Sigma	clone 1G10.2
ChaT	Goat	1/200	Millipore Sigma	AB144P
PKCα	Mouse	1/300	Santa Cruz	SC8393
Ribeye	Mouse	1/500	BD Biosciences	612044
YWHAB	Rabbit	1/200	Santa Cruz	sc-628 (discontinued)
YWHAG	Rabbit	1/200	Santa Cruz	sc-731 (discontinued)

For human samples, slides containing retinal sections were thawed in a humid chamber for approximately 10 min. To remove the OCT, they were washed with 1× PBS (three washes of 10 minutes each). Permeabilization and blocking was carried out for 1 hour using PBSGT 0.5%. Sections were next incubated overnight at room temperature and in a humid chamber with primary antibody (H3P, 1:1000, Cell Signaling no. 9701) diluted in PBSGT 0.5%. Afterward, the slides were washed again with PBSGT 0.5% (three washes of 10 minutes each), and incubated for 2 hours at room temperature, protected from light with a secondary antibody [donkey anti-rabbit IgG H&L (Alexa Fluor 555), 1:500, Abcam no. ab150062]. The secondary antibody was subsequently washed with PBST 0.5%, followed by a brief incubation with 4′,6-diamidino-2-phenylindole (DAPI; stock solution 10 mg/ml) diluted 2500 times. A final wash with 1× PBS is performed. The slides were then mounted with a coverslip using mounting media, Mowiol, and stored protected from light until imaging. Image stacks were acquired using an Olympus FV3000 confocal microscope with high-sensitivity GaAsP detectors. DAPI and Alexa Fluor 594 were excited by 405- and 559-nm laser diodes, respectively. The objective used was an Olympus UPLXAPO 20× and 40×. Microscope control and image acquisition were done via Olympus Fluoview software (FV31S Version 2.61.243) at a resolution of 1024 × 1024 pixels and a scan rate of 4 μs/pixel.

### Hematoxylin/eosin/Luxol fast-blue staining

Twenty-micrometer brain cryosections were postfixed in 10% formaldehyde for 3 hours at room temperature, then washed in PBS before incubation in Luxol Fast Blue solution [Solvan Blue 38 (10 mg/ml; Sigma-Aldrich, S3382) and 0.05% glacial acetic acid, in 95% EtOH] for 3 hours at 60°C. After cooling the excess of stain was removed in 95% ethanol (EtOH) followed by water washes, then hematoxylin staining by following: One dip in reducer solution [hydroquinone (10 mg/ml; Sigma-Aldrich, H9003) and sodium sulfite (50 mg/ml; Thermo Fisher Scientific, S-447) in water], several water washes, 10-min incubation in Gill 3 Hematoxylin solution (Stat Lab, SL200), several water washes, one dip in acid alcohol (1% HCl in 70% EtOH), several water washes, one dip in NaOH 0.05%, several water washes, and 1-min incubation in 95% EtOH. Then, eosin staining was as follows: Eosin-Y solution (Stat Lab, SL101) incubation during 1.5 minutes, 95% EtOH immersion during 3 minutes, dehydration in absolute EtOH during 3 minutes twice, followed by xylene immersion before mounting in Mowiol (Calbiochem, 475904).

### EdU pulse, EdU/Brdu dual pulse, and birth dating experiment

EdU (Abcam, ab146186) was diluted in PBS and injected intraperitoneally (ip) in pregnant mice at 50 mg/kg final concentration. For progenitor count and localization measurements, the pregnant mice were euthanized 30 min after EdU pulse, the embryos heads were harvested and directly immersed in room temperature 4% PFA diluted in PBS. For EdU/BrdU dual pulse, EdU pulse was administered in pregnant mice at E14.5 gestational day (25 mg/kg ip final concentration), then BrdU was administered 24 hours later at E15.5 gestational day (50 mg/kg ip final concentration). One hour later, the embryos heads were harvested and directly immersed in room temperature 4% PFA diluted in PBS. For birth-dating experiments EdU pulse was administered either in pregnant mice at E14.5 gestational day (25 mg/kg ip final concentration) or to P0 littermates (5 to 10 μl of 7.5 mg/ml ip concentrated EdU) then mice retinas were harvested at postnatal day P21. EdU staining was done using the EdU Click-IT reaction kit. In brief, classical immunostaining was performed first when colabeling was needed. Then, sections were washed four times in PBS and incubated with homemade EdU Click-IT solution for 15 min [PBS, 1 M ascorbic acid, 0.2 M CuSO4•5H2O, 1 mM Alexa Fluor 647 Azide (Invitrogen, A10277)]. Then sections were washed in PBS three times before mounting in Mowiol (Calbiochem, 475904100GM).

### scRNA-seq experimental procedure

E14.5 or E17.5 littermate heads were harvested and stored in Hibernate-E media (Thermo Fisher Scientific, A1247601) supplemented with B-27 (Thermo Fisher Scientific, 17504044) (2%) and GlutaMAX (1%) (Thermo Fisher Scientific, 35050061) for around 3 hours during genotyping. E14.5 cortices and E17.5 retinas from dHET and dKO littermates were then dissected into HibernateTM-E media before dissociation in papain solution [NaOH (0.05%), papain (10 U/ml), deoxyribonuclease (0.004%), and l-cysteine, in DPBS] during 2 to 4 min at 37°C. Then mechanical dissociation was done adding Lo Ovomucoid solution and flushing cells in 1-ml pipette tips. Eventually cells were filtered with a 60-μm filter and counted to resuspend them at 1000 cell/μl in PBS and 0.04% BSA. RNA library was generated using Chromium Next GEM Single Cell 3′ Reagent Kits version 3.1 Dual Index (10x Genomics, Pleasanton, CA). Note that the dataset of E14.5 cortical cells corresponds to the integration of two independent experiments from 97,675 isolated cells and 3156 isolated cells. E17.5 retinal cells correspond to a single experiment from 7156 isolated cells.

### scRNA-seq analysis

Reads were aligned on the mm10-2020-A mouse reference genome using the default parameters of Cell Ranger version 6.1.1 or later (10X Genomics) to generate unique molecular identifier counts for each gene across the cells. Average reads and genes per cell are reported in the following Table 3. Raw feature-barcode matrices from Cell Ranger count were imported in R package Seurat (version 4.1.3) for downstream analyses. Each experiment was first examined individually to identify and filter out empty/dead cells or doublets based on the number of transcripts and number of unique genes present in each cell and also on the percentage of mitochondrial reads.

**Table 3.** Average reads and genes per cell observed in scRNA-seq datasets produced for this study.

Experiment	dHET	dHET	dKO	dKO
	Mean reads/cell	Median genes/cell	Mean reads/cell	Median genes/cell
E14.5 cortex (1st experiment)	3725	758	3896	816
E14.5 cortex (2nd experiment)	51,905	1333	52,054	2636
E17.5 retina	26,120	2098	31,115	2337

Individual filtered samples were normalized, scaled, and 3000 highly variable genes identified using the SCTransform method. All four samples were then integrated using Seurat’s anchor-based integration approach. Linear dimensionality reduction performed on the integrated object by principal components analysis. Only the first 20 principal components were selected for subsequent analyses. Integrated datasets were then transferred in H5ad format for further analysis using Scanpy software (*76*) on Python. The Retina dataset was directly analyzed using Scanpy for cell quality assessment, principal components analysis, and dimensionality reduction using the uniform manifold approximation and projection (UMAP) algorithm. Unbiased clustering of single cells was performed using the Leiden algorithm. Differential gene expression analysis was performed using the *t* test with overestimated variance method. UMAP was applied for visualization purposes (reduction: pca and dims: 1:20).

### In vivo retinal electroporations and basal progenitor counts

In vivo electroporations were performed as previously described (*77*). P0 animals were anesthetized on ice and eyes were injected in the subretinal space with 1 μl of a solution of plasmid DNA (3 μg/μl). The head of the animal was then electroporated using the following setting: five pulses, 80 mV, pulse length of 50 ms, and 950-ms intervals with unipolar polarity. Eyes were collected and fixed for 30 min in 4% PFA on ice 48 hours after electroporation. To define an unbiased criteria for basal progenitor identification, we segmented the retinal section into four equal quarters and triple positive cells for SOX2/CHX10/GFP with nucleus inside the most basal quarter were counted as basal progenitors.

### Immunoprecipitation followed by MS (liquid chromatography–MS/MS)

IP-MS experiments were conducted as previously reported (*78*). Proteins were extracted in IpH buffer [tris 50 mM (pH 8.0)/150 mM NaCl/0.1% NP-40/1X Complete (Roche)/1 mM Na_3_VO_4_/5 mM EDTA] from dHET and dKO P0 retinas or cortex. Three micrograms of anti-PAR3 antibody or rabbit IgG was incubated with 30 μl of beads (Dynabeads Protein A, Invitrogen 10001D) for 2 hours at 4°C then washed in PBS 0.05% Tween. Then 1.2 μg of protein was added for overnight incubation at 4°C. After four washes in the IpH buffer and five washes in 50 mM ammonium bicarbonate.

Beads were then resuspended in 50 μl of fresh 50 mM ammonium bicarbonate. The on-bead proteins were diluted in 2 M urea/50 mM ammonium bicarbonate, and trypsin digestion was performed overnight at 37°C. The samples were reduced with 13 mM DTT at 37°C for 30 min and, after cooling for 10 min, alkylated with 23 mM chloroacetamide at room temperature for 20 min in the dark. The supernatants were acidified with trifluoroacetic acid and cleaned from residual detergents and reagents with MCX cartridges (Waters Oasis MCX 96-well Elution Plate) following the manufacturer’s recommendations. After elution in 10% ammonium hydroxide/90% methanol (v/v), samples were dried with SpeedVac, reconstituted under agitation for 15 min in 12 μl of 2% acetonitrile (ACN)–1% formic acid (FA), and loaded into a 75 μm inside diameter × 150-mm Self-Pack C18 column installed in the Easy-nLC II system (Proxeon Biosystems). Peptides were eluted with a two-slope gradient at a flow rate of 250 nl/min. Solvent B (0.2% FA in ACN) first increased from 2 to 35% in 105 min and then from 35 to 85% B in 15 min. The high-performance liquid chromatography system was coupled to an Orbitrap Fusion mass spectrometer (Thermo Fisher Scientific) through the Nanospray Flex Ion Source. Nanospray and S-lens voltages were set to 1.3 to 1.7 kV and 50 V, respectively. Capillary temperature was set to 225°C. Full-scan MS survey spectra [mass/charge ratio (*m/z*) 360 to 1560] in profile mode were acquired by the Orbitrap with a resolution of 120,000 with a target value at 3 × 105. The 25 most intense peptide ions were fragmented in the high collision dissociation (HCD) cell and analyzed in the linear ion trap with a target value at 2 × 104 and a normalized collision energy at 28 V. Target ions selected for fragmentation were dynamically excluded for 25 s after two MS/MS events. The peak list files were generated using Proteome Discoverer program (version 2.3) with the following parameters: minimum mass set to 500 Da, maximum mass set to 6000 Da, no grouping of MS/MS spectra, precursor charge set to auto, and minimum number of fragment ions set to 5. Protein database search was performed using the Mascot 2.6 program (Matrix Science) against the UniProt *Mus musculus* protein database. The mass tolerances for precursor and fragment ions were set to 10 parts per million and 0.6 Da, respectively. Data interpretation was performed using Scaffold program (version 4.10.0). Data analysis was done on Excel (Microsoft), considering exclusive spectrum counts and applying the following filters: All the hits with a minimum of five exclusive spectrum counts and enriched fivefold over IgG control were considered.

### Quantitative analysis

#### EdU counts and localization

EdU^+^ progenitor number and localization were semi-automatically measured using homemade ImageJ macro. In brief, images were filtered using the fast Fourier transform bandpass filter to smooth EdU staining and help EdU^+^ nuclei segmentation. Then EdU^+^ cells were selected using manual threshold to select EdU staining. Overlapping EdU^+^ nuclei were separated using the ImageJ Watershed function and the Analyze particles function of ImageJ were used to select the EdU^+^ nuclei. Then, each EdU^+^ nucleus relative distance from apical and basal border of the cortex or retina is calculated using geodesic distance map function of MorphoLibJ (*79*) and expressed as percentage distance from apical border. To ensure consistency of the region analyzed between samples, the optic nerve was used as a landmark for central position in the developing retina, and the anterior commissure was used as a landmark for central position in the developing cortex. Data are quantified as counts per unit apical/ventricular length (reported as number of cells per micrometer) rather than per square micrometer. Because tissue thickness varies substantially between conditions at E14.5, making areal normalization (cells/μm^2^) would have been misleading for comparisons of total cell number.

#### Cell type counts

The number of cells expressing the different cell type–specific markers was quantified by averaging the number of positive cells from two to three regions of at least 350 μm in the retina and large tile scan area of the cortex using hippocampus as anatomical landmark. Cells were either counted manually or using Stardist deep-learning algorithm on ImageJ software when markers were nuclear (*80*). The markers used to identify and count each cell population are the one presented in the representative images within the figures except for Rods where the very particular nuclear inverted chromatin pattern was used to count rod photoreceptor nuclei. Cell numbers were normalized by the total number of cells counted in the retina since all cell types were counted for each sample. For cortex counts, normalization was made over the total number of nuclei counted on Hoechst staining to ensure no cells were omitted. For statistical comparisons, cells from all normal layers were counted in the retina (ONL-INL-GCL) for both dHET and dKO samples and values were compared with dKO extra-layer proportions. Similarly in the cortex, dHET and dKO proportions were compared to the subcortical heterotopia cell types proportions. To ensure consistency of the regions analyzed between sample landmarks were used. The optic nerve was used as a landmark for central position in the retina. The hippocampus was used as a landmark for position in the cortex.

#### YAP1/TAZ ratio

After cryosectioning, YAP1/TAZ immunostaining of dHET and dKO developing tissues, and image acquisition using a confocal microscope, images were analyzed using Fiji by drawing equally sized region of interest (ROI) at two different positions of the apical and basal side of the tissue. Mean fluorescence intensity of each ROI is then averaged and the ratio between mean fluorescence intensity in the apical and basal side of the tissue is compared between dHET and dKO embryos. All statistics were done using Graphpad Prism software version 8.4.3

## Supplementary Materials

### The PDF file includes:

Figs. S1 to S13 Legends for tables S1 and S2 References
