# Supplementary material for: Oriented cell divisions induce basal progenitors and regulate neural expansion across tissues and species
Source: Sci Adv. 2026 Feb 4;12(6):eadz6827. doi: 10.1126/sciadv.adz6827 (PMC12871468; doi:10.1126/sciadv.adz6827)
Supplement: Supplementary file 1 — Figs. S1 to S13 Legends for tables S1 and S2 References [file sciadv.adz6827_sm.pdf]

Science Advances
AAAS

Supplementary Materials for

Oriented cell divisions induce basal progenitors and regulate neural expansion across tissues and species

Benoit Boulan *et al.*

Corresponding author: Michel Cayouette, michel.cayouette@ircm.qc.ca

Sci. Adv. **12**, eadz6827 (2026)
DOI: 10.1126/sciadv.adz6827

The PDF file includes:

Figs. S1 to S13
Legends for tables S1 and S2
References

Other Supplementary Material for this manuscript includes the following:

Tables S1 and S2

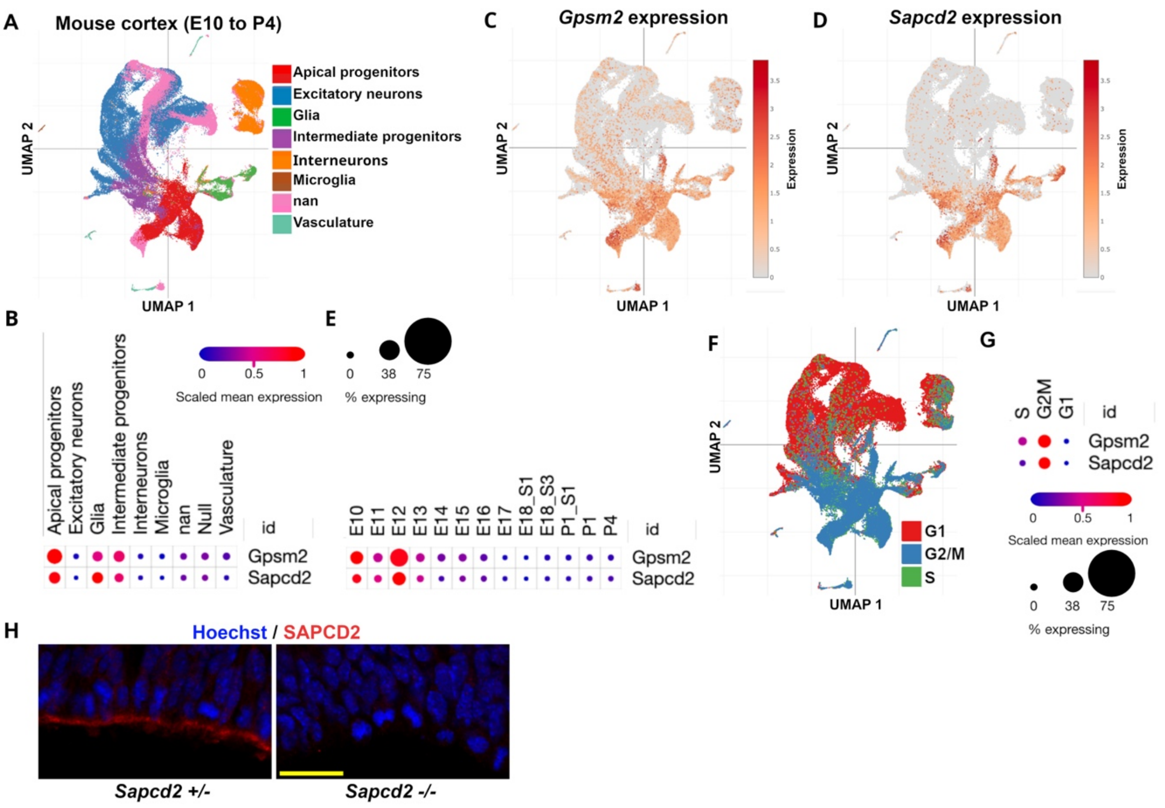

FIGURE S1. Single-cell RNA sequencing of *Gpsm2* and *Sapcd2* expression in developing cortex and retina.

(A) UMAP of cortical cell type clusters identified in all cells combined from E10.5 to P4 timepoints from Di Bella et al., 2021 (*81*) using the Broad Institute's single-cell portal. Nan cluster refers to unassigned cell types. **(B)** *Gpsm2* and *Sapcd2* expression levels by cell types showing enrichment in apical progenitors, intermediate progenitors, and glial cells. **(C-D)** UMAP illustrating *Gpsm2* (C) and *Sapcd2* (D) expression levels. **(E)** *Gpsm2* and *Sapcd2* expression levels at different timepoints. (**F**) UMAP of cell cycle states enrichment by cell types. (**G**) *Gpsm2* and *Sapcd2* expression levels as a function of cell cycle phases. (**H**) Immunostaining for SAPCD2 (red signal) in *Sapcd2*$^{-/-}$ and *Sapcd2*$^{+/-}$ E14.5 embryonic cortex. Staining shows loss of signal in Sapcd2$^{-/-}$ and clear enrichment at apical domain of RGCs in Sapcd2$^{+/-}$, similar to the expression pattern previously observed in retina (*24*). Scale bar: 20 µm.

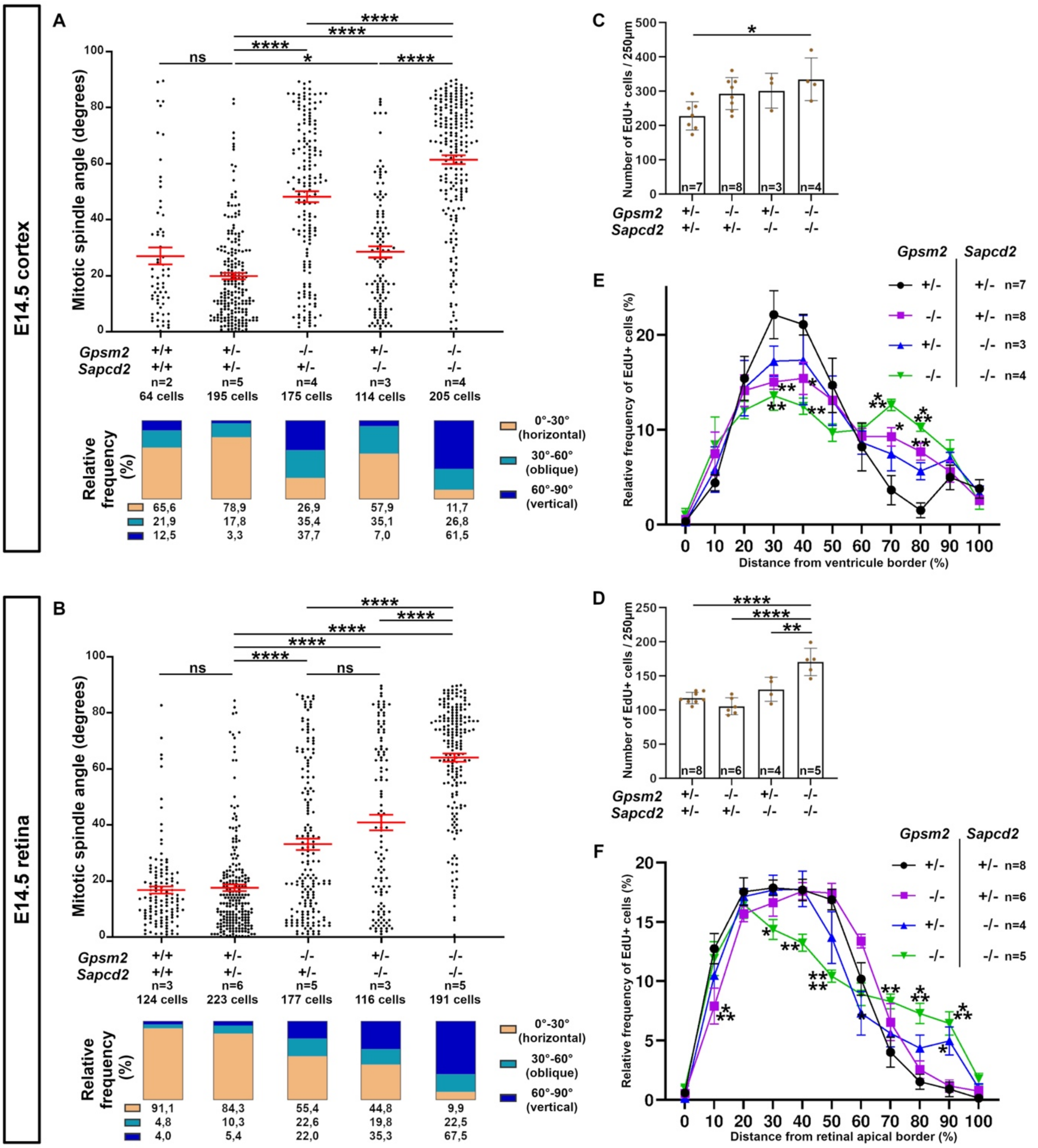

FIGURE S2. ***Gpsm2* and *Sapcd2* cooperate to regulate spindle orientation, progenitor number and basal relocalisation.**

(A-B) Quantification of the mitotic spindle orientation of E14 cortex (A) and retina (B). Each dot represents the orientation of a dividing progenitor. Bars represent medians. Histograms of mitotic spindle angles relative frequency represented in 3 bins (horizontal/oblique/vertical) for each genotype. **(C-D)** Quantification of the total number of EdU+ dividing progenitors in E14.5 cortex (C) and E14.5 retina (D) after 1h pulse of EdU. Comparison between groups was done using a 1 way ANOVA test followed by Tukey's correction for multiple comparisons. **(E-F)** Quantification of EdU+ cell localization relative to the apical surface of cortex (E) and retinal tissue (F). Distance between EdU+ cells and apical border of the tissue is measured and

expressed as a percentage of the tissue thickness. Note the correlation between division reorientation and basal relocalisation of EdU+ cells. Comparison between groups was done using a 2-way ANOVA test followed by Dunnett's correction for multiple comparisons. (* = p < 0.05, ** = p < 0.01, *** = p < 0.001, **** = p < 0.0001). Scale bars: 100µm.

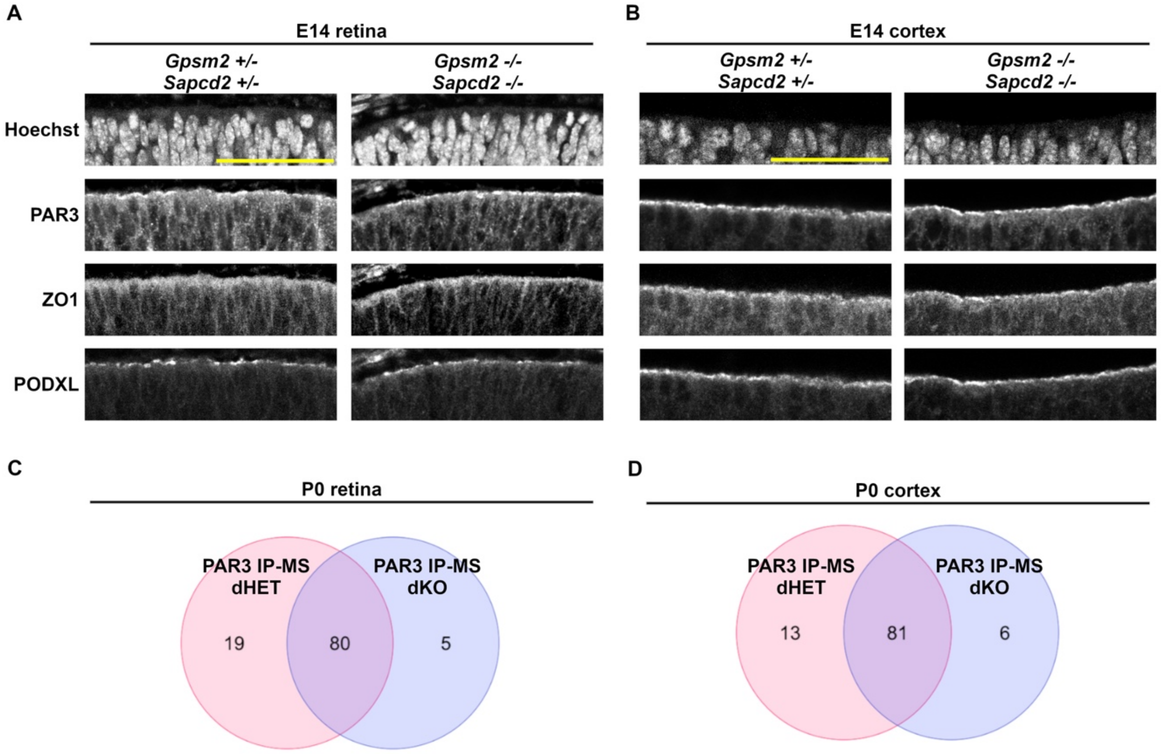

FIGURE S3. *Gpsm2/Sapcd2* dKO retina and cortex have normal polarity.
(A-B) Immunostainings for the polarity protein PAR3 and the apical membrane markers ZO1 and PODXL show no change in dKOs compared to double heterozygous controls in the E14 retina (A) and cortex (B). Scale bars: 50 µm **(C-D)** Venn Diagram of protein found by IP-MS to interact with PAR3 in P0 retina (C) and cortex (D). Spectral count threshold is set at a minimum of 5 for more stringency.

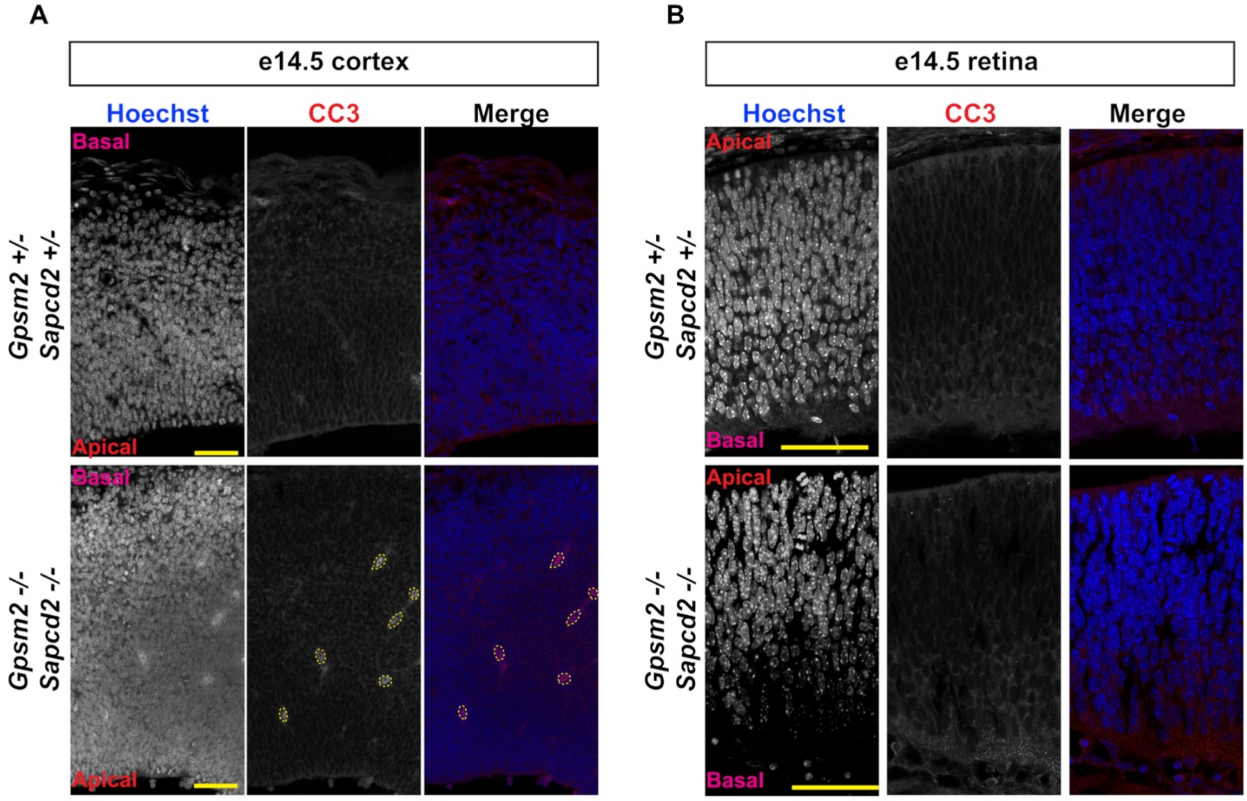

FIGURE S4. *Gpsm2/Sapcd2* dKO induces few apoptosis in developing cortex but none in developing retina. Representative images of dHET and dKO E14.5 cortical (A) and retinal (B) sections stained for Cleaved Caspase 3 (CC3).

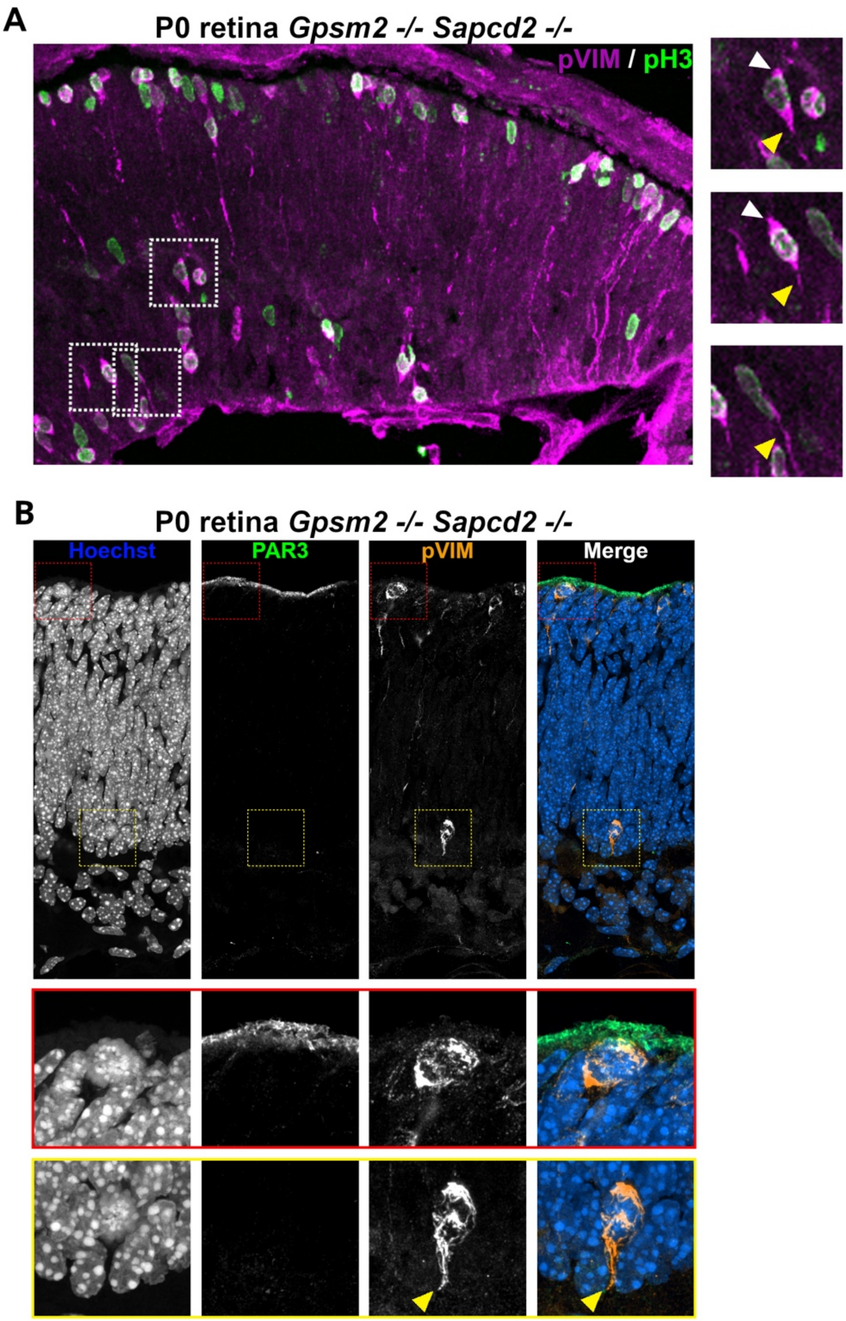

FIGURE S5. *Gpsm2/Sapcd2* dKO retinas feature unipolar and bipolar basal progenitors. (A) Immunostaining for the progenitor marker phospho-vimentin (pVIM) and the mitotic cell marker phospho-histone 3 (pH3) on P0 retinal sections from Gpsm2/Spacd2 dKO. White boxed areas are shown at higher magnification on the right. The white arrowheads point to the apical pole lacking a process, whereas the yellow arrowheads point to the basal process. (B) Immunostaining for pVIM and the apical membrane marker PAR3 on P0 retinal sections from Gpsm2/Spacd2 dKO. The boxed areas on the top panels are shown at higher magnifications in the bottom panels. One cell undergoes mitosis in contact with the PAR3+ apical surface (red box), whereas the other cell undergoes mitosis on the basal side of the neuroepithelium and still maintains a basal process (yellow arrowhead) but no apical attachment (yellow box).

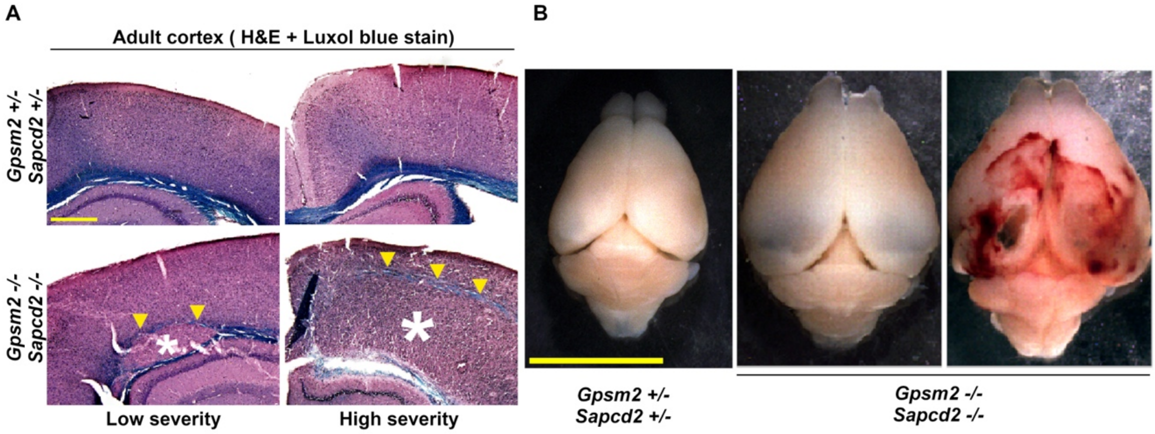

FIGURE S6. *Gpsm2/Sapcd2* dKO induces subcortical heterotopia and haemorrhagic hydrocephalus with variable severity.
(A) Representative images of adult cortices colored by Hematoxylin & Eosin in addition to Luxol fast blue. Luxol fast blue stains the white matter, enabling the delineation of subcortical heterotopia bands (yellow arrowheads). In both examples of dKO adult brains, note the variability in subcortical heterotopia band size (white stars). Scale bar: 500µm. **(B)** Representative pictures of dHET (left panel) and two dKO adult brains (middle and right panel). Note that dKO brains appear visually larger than dHET. Variability in phenotype leads in the worst cases to hemorrhagic hydrocephalus (right panel), Scale bar: 1 cm.

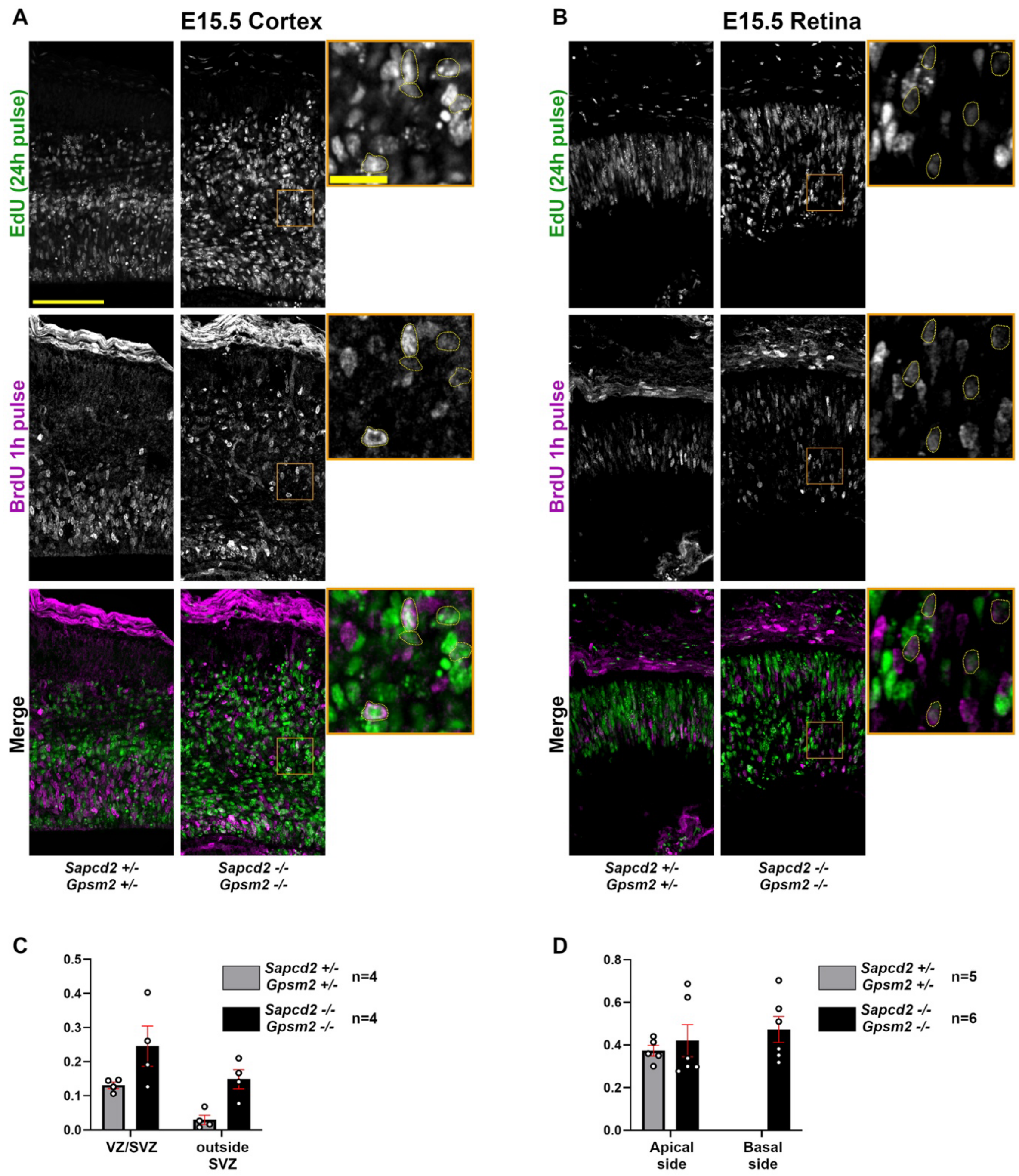

Figure S7. Basal progenitors re-enter the cell cycle more frequently in the *Gpsm2/Sapcd2* dKO cortex and retina. Cortical (A) and retinal (B) sections at E15.5 stained for EdU and BrdU after administration of EdU at E14.5 and BrdU 24 h later, 1 h before tissue collection. Many cells on the basal side of the neuroepithelium (boxed area, zoom-in shown on the right) are double-positive, indicating re-entry in the cell cycle. (C, D) Bar graphs showing the quantification of the ratio of double positive cells over all EdU+ cells (EdU+BrdU+/EdU+) in the VZ/SVZ and outside the SVZ in the cortex (C) and on the apical and basal side of the epithelium in the retina (D) in dHET and dKO. The proportion of double-positive cells outside the cortical SVZ or on the basal part of the retina is higher in dKO than dHET controls.

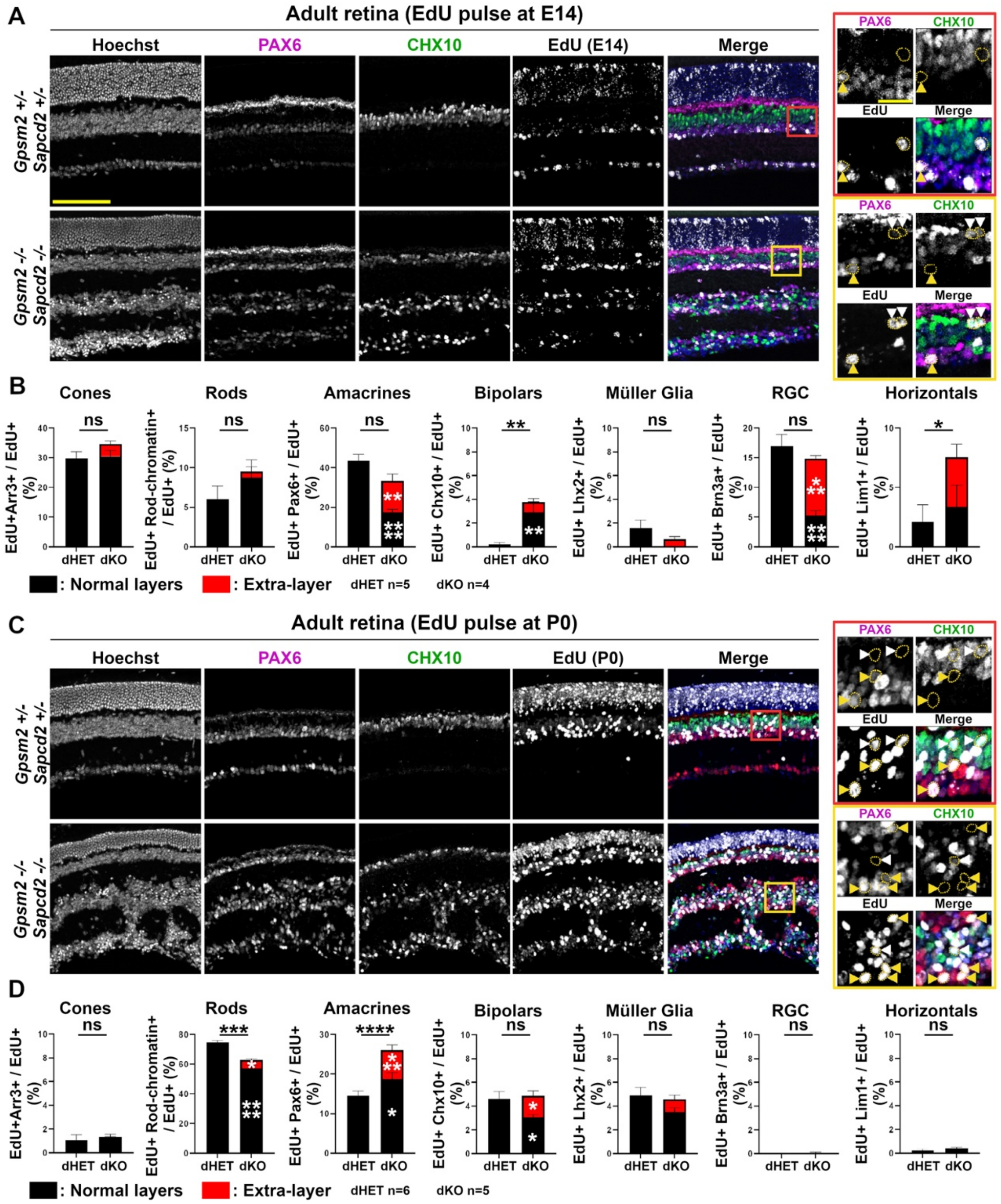

FIGURE S8. Temporal patterning is altered in dKO retinas.

(A) Representative images of P21 retina after an EdU pulse administered at E14. Co-immunolabeling of bipolar cells (CHX10), amacrine cells (PAX6), and EdU. Zoom-in panels on the right shows the absence of EdU+/CHX10+ double positive cells in dHET, whereas they are readily observed in dKO samples (white arrowheads). **(B)** Quantification of retinal cell type birthdate by the number of EdU+/marker+ ratios for each cell type. **(C)** Representative images of P21 retina following EdU pulse administered at P0. Co-immunolabeling of bipolar cells (CHX10), amacrine cells (PAX6), and EdU. Zoom-in panel on the right shows the increase of amacrine cell number produced at late developmental stage as reflected by the high number

of EdU+/PAX6+ double positive cells in dKO sample compared to dHET (yellow arrowheads). **(D)** Quantification of retinal cell birthdate by the number of EdU+/marker+ ratios for each cell type. Scale bars: 100 µm (upper panels), 20 µm (zoom-in). All comparisons between groups were done using a 2-way ANOVA test followed by Sidak's correction for multiple comparisons. (* = $p < 0.05$, ** = $p < 0.01$, *** = $p < 0.001$, **** = $p < 0.0001$).

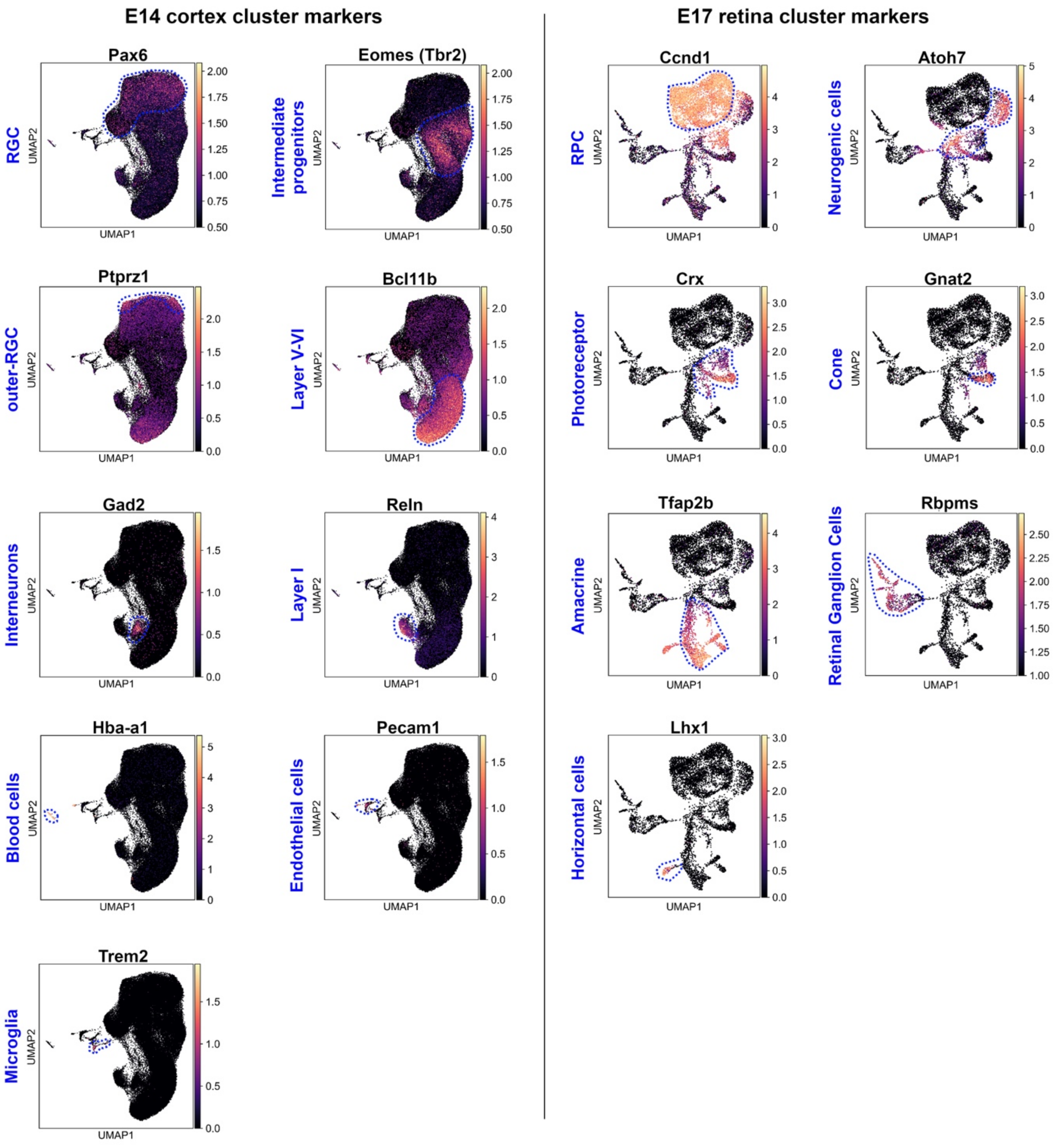

FIGURE S9. Single cell RNA sequencing of embryonic dHET and dKO cortex and retinas. Examples of cell-type specific marker expression in the different cell clusters.

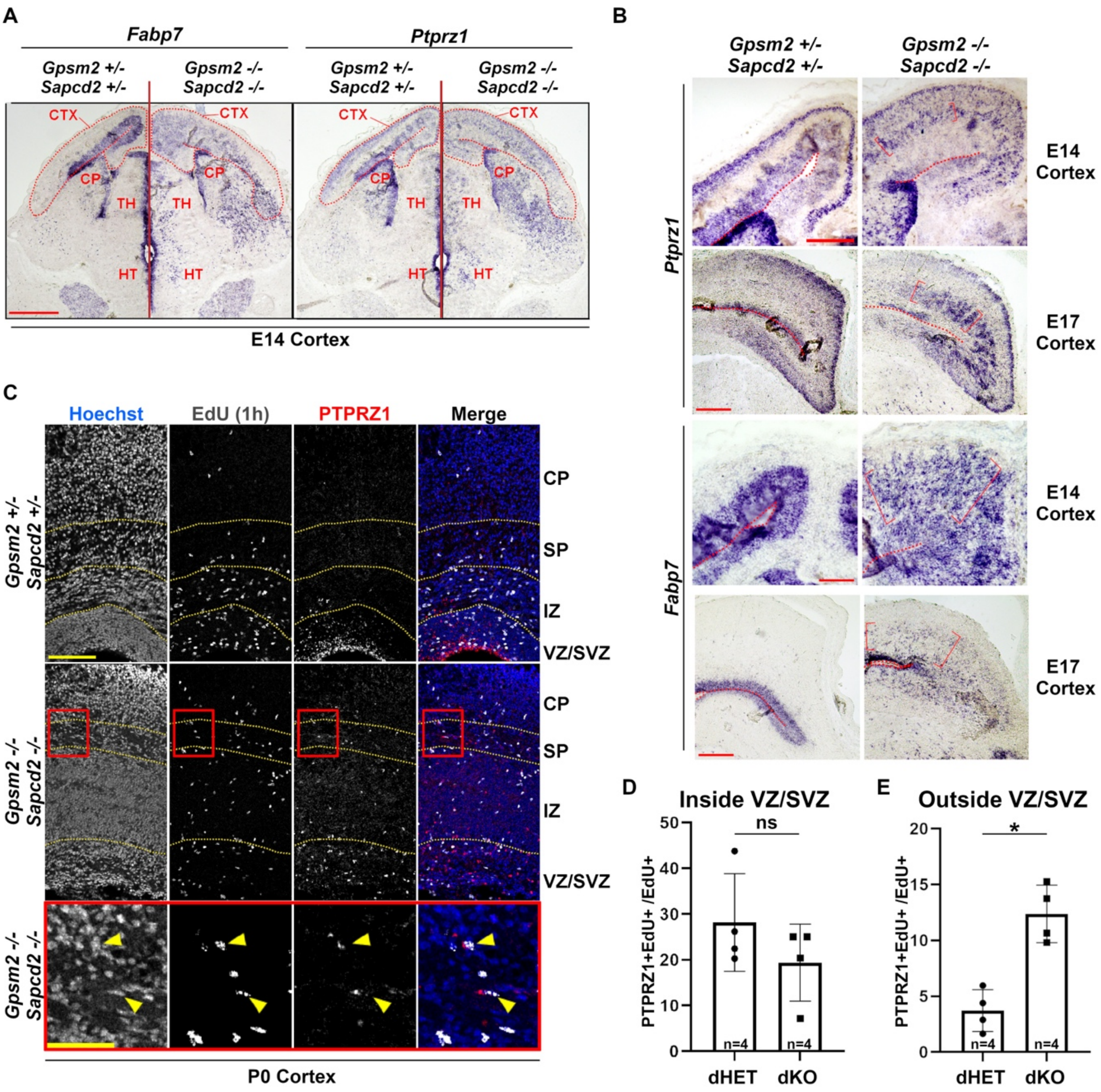

FIGURE S10. ***Gpsm2/Sapcd*2 dKO cortices have more PTPRZ1+ oRGCs.**
(A, B) In situ hybridization for *Fabp7* and *Ptprz1* mRNA shows an increase in basally located cells expressing these markers in the dKO mouse cortex. Low magnification images (A) show the general increase of *Fabp7* and *Ptprz1* expression in E14 dKO brains. High magnification images (B) of the E14 and E17 neocortex show the increase in basally located cells expressing *Fabp7* and *Ptprz1* mRNA (red brackets). Red dashed lines outline the ventricular border. Scale bars: 500 µm (panel A), 200 µm (panel B). CTX=Cortex, CP=Caudate Putamen, HT=Hypothalamus, TH=Thalamus. **(C)** Co-immunostaining for PTPRZ1 and EdU after a 30-minute pulse at P0. The bottom row of images show magnified view of the red boxed areas. **(D-E)** Quantification of PTPRZ1+/EdU+ double positive cells inside (D) or outside (E) the VZ/SVZ in dKO and dHET. Scale bars: 100 µm (upper panels), 50 µm (zoom-in). Comparison between groups was done using Mann Whitney test (* = $p < 0.05$). VZ=Ventricular zone, SVZ=Subventricular zone, IZ=Intermediate zone, SP=Subplate, CP=Cortical plate.

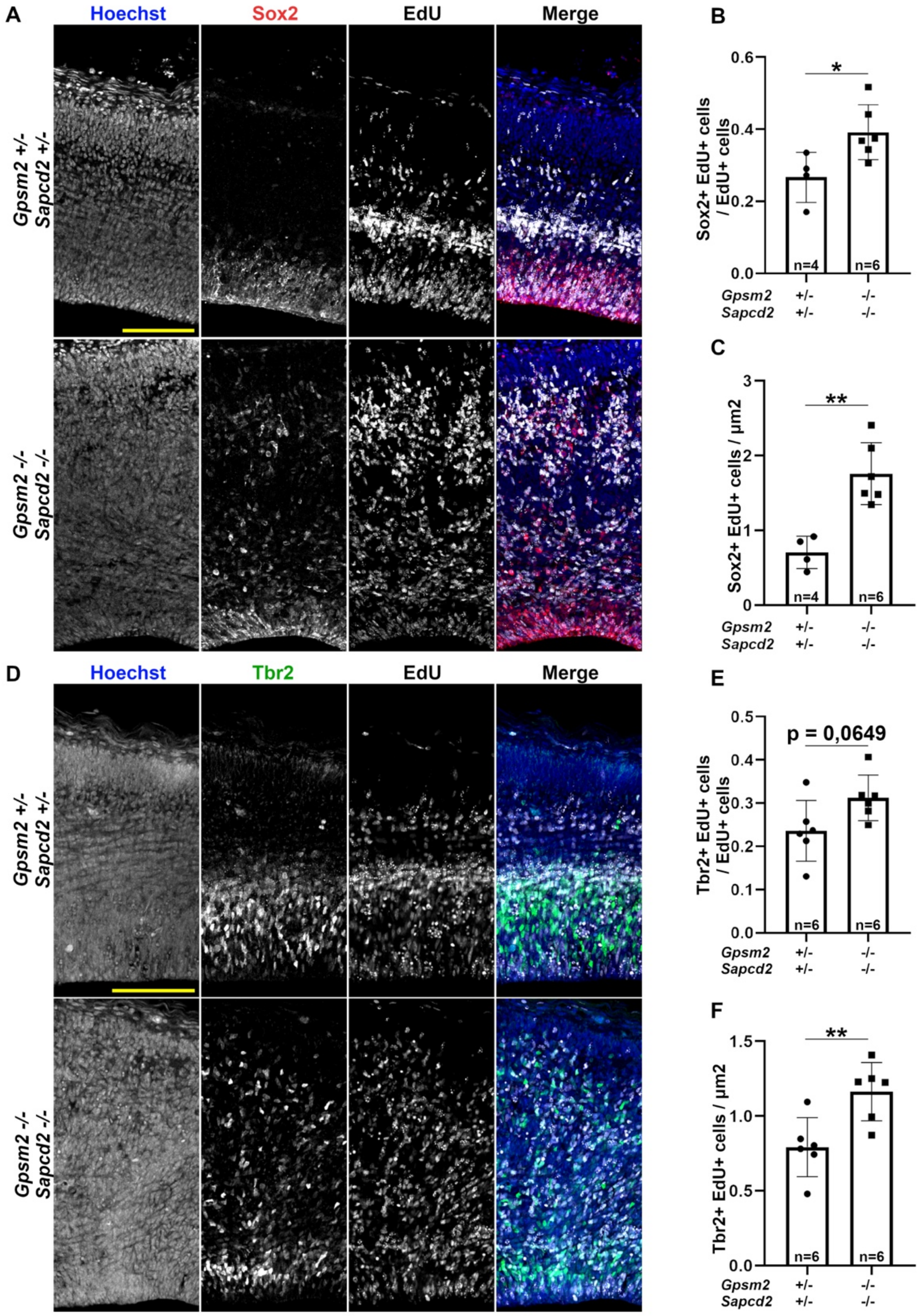

FIGURE S11. The proportion of RGCs and the total number of RGC and IP is increased in the *Gpsm2/Sapcd2* dKO neocortex.

(A) Co-immunostaining for SOX2 and EdU on E14.5 neocortex sections 24 hours after administration of EdU. Scale bar: 100 µm. **(B)** Quantification of SOX2+/EdU+ double positive cell proportion normalized by total EdU+ cells in the same area. **(C)** Quantification of absolute

SOX2+/EdU+ double positive cell number normalized by region of interest (ROI) size. **(D)** Co-immunostaining for TBR2 (EOMES) and EdU on E14.5 neocortex sections 24 hours after administration of EdU. Scale bar: 100 µm. **(E)** Quantification of TBR2+/EdU+ double positive cell proportion normalized by total EdU+ cells in the same area. **(F)** Quantification of absolute TBR2+/EdU+ double positive cell number normalized by ROI area size. Comparison between groups was done using Mann Whitney test (* = $p < 0.05$, ** = $p < 0.01$).

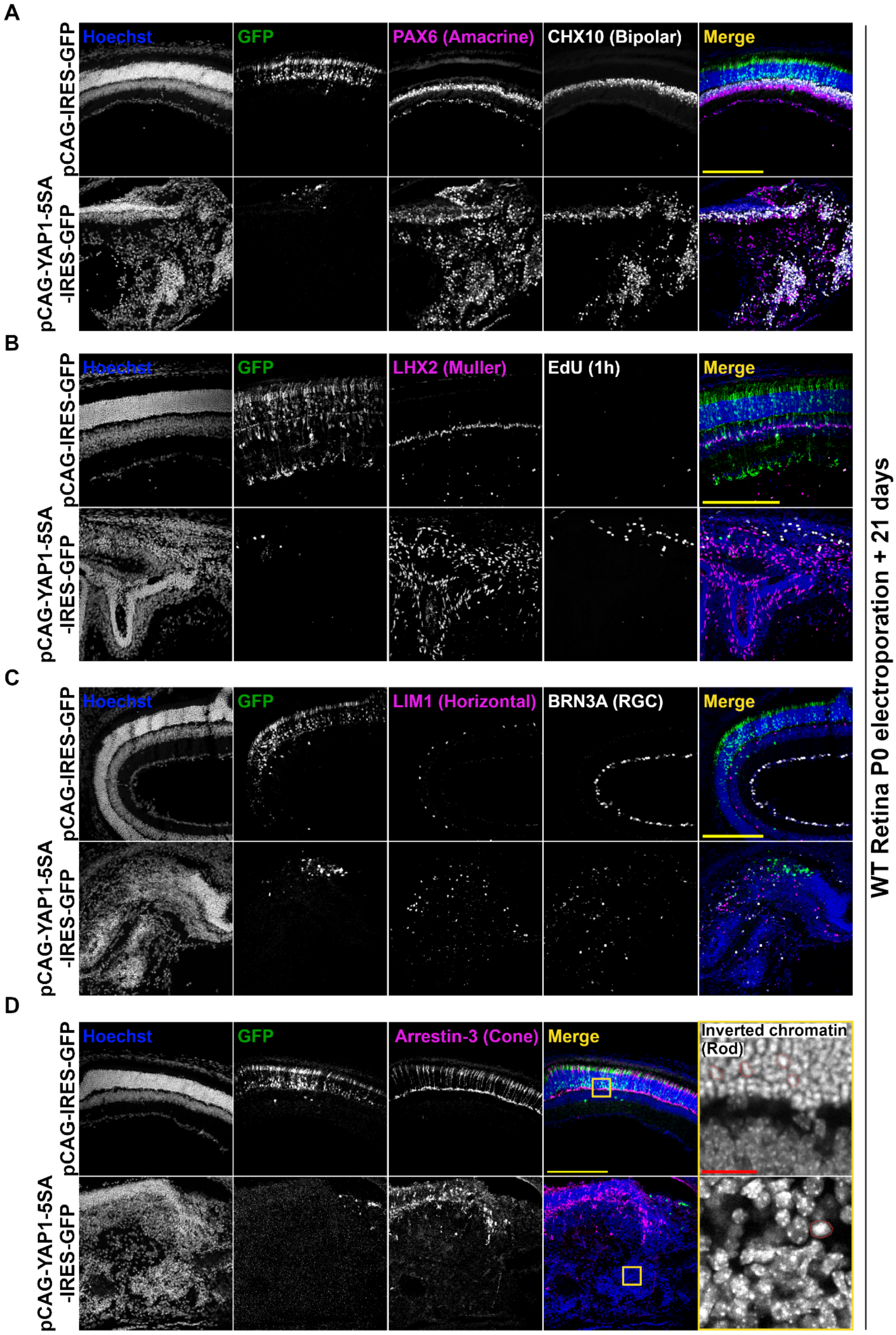

FIGURE S12. Hippo pathway activation tends to generate amacrine, bipolar and Müller glia cell types. Co-immunolabeling for GFP and various cell type specific markers, as

indicated, 21 days after P0 retinal electroporation of either pCAG-IRES-GFP or pCAG-YAP1-5SA-IRES-GFP plasmids. Representative images of 3 independent WT retinas electroporated.

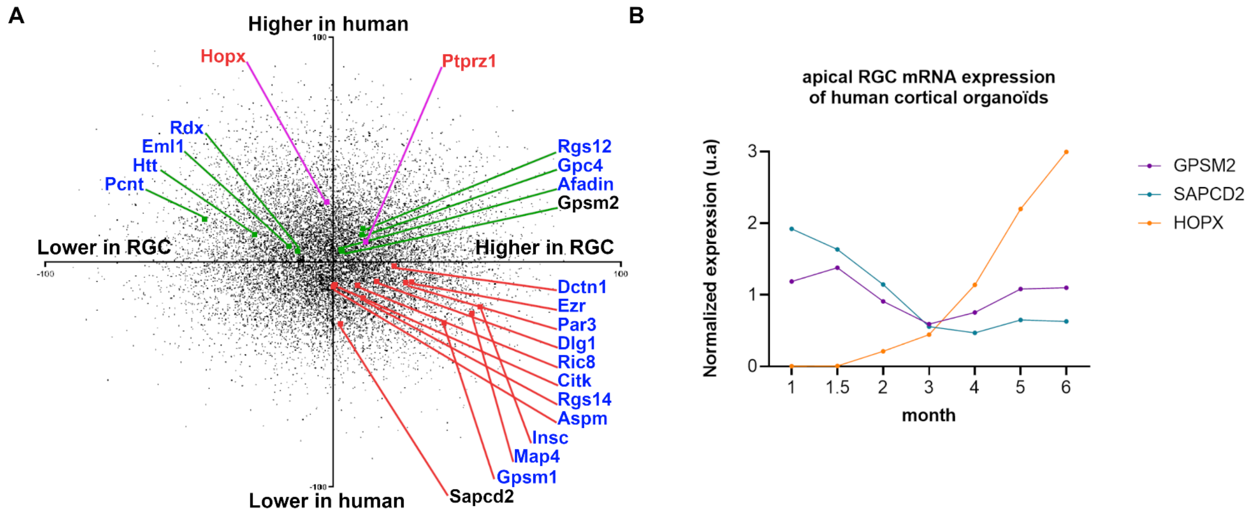

FIGURE S13. Mitotic spindle orientation regulators are downregulated in the human compared to the mouse embryonic brain. (A) Dot plot representing the differential expression of 15,576 homologous genes between human and mouse developing brains from Lui et al. 2014 (*57*). The x axis represents RGC specificity based on human and mouse differential expression of homologous genes between RGCs and other developing brain cell types. The y axis represents differential expression of homologous genes between humans and mice. Genes in red are oRGCs markers, genes in blue are known regulators of mitotic spindle orientation. **(B)** Human cortical organoids normalised mRNA levels of *Gpsm2* (purple), *Sapcd2* (blue) and *Hopx* (orange) at different stages of culture from Uzquiano, Kedaigle et al. 2022 (*58*).

Supplement TABLE S1. IP-MS analysis of PAR3 interacting partners in P0 dHET versus P0 dKO retina and cortex.

Supplement TABLE S2. Differentially expressed genes of E14.5 mouse oRGC and comparison with human oRGC differentially expressed genes.

REFERENCES

1. S. Bizzotto, F. Francis, Morphological and functional aspects of progenitors perturbed in cortical malformations. *Front. Cell. Neurosci.* **9**, 30 (2015).

2. T. Namba, W. B. Huttner, Neural progenitor cells and their role in the development and evolutionary expansion of the neocortex. *Wiley Interdiscip. Rev. Dev. Biol.* **6**, e256 (2017).

3. D. V. Hansen, J. H. Lui, P. R. L. Parker, A. R. Kriegstein, Neurogenic radial glia in the outer subventricular zone of human neocortex. *Nature* **464**, 554–561 (2010).

4. S. Mayer, A. R. Kriegstein, "The expansion of the cortical sheet in primates" in *Evolutionary Neuroscience* (Elsevier, 2020), pp. 519–532.

5. J. L. Fish, C. Dehay, H. Kennedy, W. B. Huttner, Making bigger brains-the evolution of neural-progenitor-cell division. *J. Cell Sci.* **121**, 2783–2793 (2008).

6. J. Liu, W. Liu, L. Yang, Q. Wu, H. Zhang, A. Fang, L. Li, X. Xu, L. Sun, J. Zhang, F. Tang, X. Wang, The primate-specific gene TMEM14B marks outer radial Glia cells and promotes cortical expansion and folding. *Cell Stem Cell* **21**, 635–649.e8 (2017).

7. M. Kostic, J. T. M. L. Paridaen, K. R. Long, N. Kalebic, B. Langen, N. Grübling, P. Wimberger, H. Kawasaki, T. Namba, W. B. Huttner, YAP activity is necessary and sufficient for basal progenitor abundance and proliferation in the developing neocortex. *Cell Rep.* **27**, 1103–1118.e6 (2019).

8. K. H. Siller, C. Q. Doe, Spindle orientation during asymmetric cell division. *Nat. Cell Biol.* **11**, 365–374 (2009).

9. B. E. LaMonica, J. H. Lui, D. V. Hansen, A. R. Kriegstein, Mitotic spindle orientation predicts outer radial glial cell generation in human neocortex. *Nat. Commun.* **4**, 1665 (2013).

10. A. Shitamukai, D. Konno, F. Matsuzaki, Oblique radial glial divisions in the developing mouse neocortex induce self-renewing progenitors outside the germinal zone that resemble primate outer subventricular zone progenitors. *J. Neurosci.* **31**, 3683–3695 (2011).

11. L. Godinho, P. R. Williams, Y. Claassen, E. Provost, S. D. Leach, M. Kamermans, R. O. L. Wong, Nonapical symmetric divisions underlie horizontal cell layer formation in the developing retina in vivo. *Neuron* **56**, 597–603 (2007).

12. I. P. Weber, A. P. Ramos, P. J. Strzyz, L. C. Leung, S. Young, C. Norden, Mitotic position and morphology of committed precursor cells in the zebrafish retina adapt to architectural changes upon tissue maturation. *Cell Rep.* **7**, 386–397 (2014).

13. S. Shirazi Fard, M. Thyselius, C. All-Ericsson, F. Hallböök, The terminal basal mitosis of chicken retinal Lim1 horizontal cells is not sensitive to cisplatin-induced cell cycle arrest. *Cell Cycle* **13**, 3698–3706 (2014).

14. M. Cayouette, Expanding neuronal layers by the local division of committed precursors. *Neuron* **56**, 575–577 (2007).

15. T. Marquardt, S. L. Pfaff, Cracking the transcriptional code for cell specification in the neural tube. *Cell* **106**, 651–654 (2001).

16. J. M. Trimarchi, M. B. Stadler, C. L. Cepko, Individual retinal progenitor cells display extensive heterogeneity of gene expression. *PLOS ONE* **3**, e1588 (2008).

17. V. A. Wallace, Concise review: Making a retina—From the building blocks to clinical applications. *Stem Cells* **29**, 412–417 (2011).

18. F. Cremisi, MicroRNAs and cell fate in cortical and retinal development. *Front. Cell. Neurosci.* **7**, 141 (2013).

19. P. L. Santos-França, L. A. David, F. Kassem, X. Q. Meng, M. Cayouette, Time to see: How temporal identity factors specify the developing mammalian retina. *Semin. Cell Dev. Biol.* **142**, 36–42 (2023).

20. M. Cayouette, M. Raff, The orientation of cell division influences cell-fate choice in the developing mammalian retina. *Development* **130**, 2329–2339 (2003).

21. A. Kechad, C. Jolicoeur, A. Tufford, P. Mattar, R. W. Y. Chow, W. A. Harris, M. Cayouette, Numb is required for the production of terminal asymmetric cell divisions in the developing mouse retina. *J. Neurosci.* **32**, 17197–17210 (2012).

22. M. Lacomme, B. Tarchini, C. Boudreau-Pinsonneault, C. Monat, M. Cayouette, The LGN protein promotes planar proliferative divisions in the neocortex but apicobasal asymmetric terminal divisions in the retina. *Development* **143**, 575–581 (2016).

23. F. di Pietro, A. Echard, X. Morin, Regulation of mitotic spindle orientation: An integrated view. *EMBO Rep.* **17**, 1106–1130 (2016).

24. C. W. N. Chiu, C. Monat, M. Robitaille, M. Lacomme, A. M. Daulat, G. Macleod, H. McNeill, M. Cayouette, S. Angers, SAPCD2 controls spindle orientation and asymmetric divisions by negatively regulating the Gαi-LGN-NuMA ternary complex. *Dev. Cell* **36**, 50–62 (2016).

25. M. Gai, F. T. Bianchi, C. Vagnoni, F. Vernì, S. Bonaccorsi, S. Pasquero, G. E. Berto, F. Sgrò, A. M. A. Chiotto, L. Annaratone, A. Sapino, A. Bergo, N. Landsberger, J. Bond, W. B. Huttner, F. Di Cunto, ASPM and CITK regulate spindle orientation by affecting the dynamics of astral microtubules. *EMBO Rep.* **17**, 1396–1409 (2016).

26. M. Kielar, F. P. D. Tuy, S. Bizzotto, C. Lebrand, C. de Juan Romero, K. Poirier, R. Oegema, G. M. Mancini, N. Bahi-Buisson, R. Olaso, A.-G. Le Moing, K. Boutourlinsky, D. Boucher, W. Carpentier, P. Berquin, J.-F. Deleuze, R. Belvindrah, V. Borrell, E. Welker, J. Chelly, A. Croquelois, F. Francis, Mutations in Eml1 lead to ectopic progenitors and neuronal heterotopia in mouse and human. *Nat. Neurosci.* **17**, 923–933 (2014).

27. M. Saadaoui, M. Machicoane, F. di Pietro, F. Etoc, A. Echard, X. Morin, Dlg1 controls planar spindle orientation in the neuroepithelium through direct interaction with LGN. *J. Cell Biol.* **206**, 707–717 (2014).

28. K. Sanada, L.-H. Tsai, G protein betagamma subunits and AGS3 control spindle orientation and asymmetric cell fate of cerebral cortical progenitors. *Cell* **122**, 119–131 (2005).

29. M. Zigman, M. Cayouette, C. Charalambous, A. Schleiffer, O. Hoeller, D. Dunican, C. R. McCudden, N. Firnberg, B. A. Barres, D. P. Siderovski, J. A. Knoblich, Mammalian inscuteable regulates spindle orientation and cell fate in the developing retina. *Neuron* **48**, 539–545 (2005).

30. E. Peyre, X. Morin, An oblique view on the role of spindle orientation in vertebrate neurogenesis. *Dev. Growth Differ.* **54**, 287–305 (2012).

31. F. Mora-Bermúdez, W. B. Huttner, Novel insights into mammalian embryonic neural stem cell division: Focus on microtubules. *Mol. Biol. Cell* **26**, 4302–4306 (2015).

32. X. Morin, F. Jaouen, P. Durbec, Control of planar divisions by the G-protein regulator ` maintains progenitors in the chick neuroepithelium. *Nat. Neurosci.* **10**, 1440–1448 (2007).

33. D. T. Bergstralh, D. St Johnston, Spindle orientation: What if it goes wrong? *Semin. Cell Dev. Biol.* **34**, 140–145 (2014).

34. R. S. Bultje, D. R. Castaneda-Castellanos, L. Y. Jan, Y.-N. Jan, A. R. Kriegstein, S.-H. Shi, Mammalian Par3 regulates progenitor cell asymmetric division via notch signaling in the developing neocortex. *Neuron* **63**, 189–202 (2009).

35. D. Konno, G. Shioi, A. Shitamukai, A. Mori, H. Kiyonari, T. Miyata, F. Matsuzaki, Neuroepithelial progenitors undergo LGN-dependent planar divisions to maintain self-renewability during mammalian neurogenesis. *Nat. Cell Biol.* **10**, 93–101 (2008).

36. Q. Du, I. G. Macara, Mammalian Pins is a conformational switch that links NuMA to heterotrimeric G proteins. *Cell* **119**, 503–516 (2004).

37. M. Cayouette, A. V. Whitmore, G. Jeffery, M. Raff, Asymmetric segregation of Numb in retinal development and the influence of the pigmented epithelium. *J. Neurosci.* **21**, 5643–5651 (2001).

38. A. Jabali, A. Hoffrichter, A. Uzquiano, F. Marsoner, R. Wilkens, M. Siekmann, B. Bohl, A. C. Rossetti, S. Horschitz, P. Koch, F. Francis, J. Ladewig, Human cerebral organoids reveal progenitor pathology in EML1-linked cortical malformation. *EMBO Rep.* **23**, e54027 (2022).

39. P. J. Strzyz, H. O. Lee, J. Sidhaye, I. P. Weber, L. C. Leung, C. Norden, Interkinetic nuclear migration is centrosome independent and ensures apical cell division to maintain tissue integrity. *Dev. Cell* **32**, 203–219 (2015).

40. N. Kalebic, W. B. Huttner, Basal progenitor morphology and neocortex evolution. *Trends Neurosci.* **43**, 843–853 (2020).

41. A. A. Pollen, T. J. Nowakowski, J. Chen, H. Retallack, C. Sandoval-Espinosa, C. R. Nicholas, J. Shuga, S. J. Liu, M. C. Oldham, A. Diaz, D. A. Lim, A. A. Leyrat, J. A. West, A. R. Kriegstein, Molecular identity of human outer radial glia during cortical development. *Cell* **163**, 55–67 (2015).

42. E. R. Thomsen, J. K. Mich, Z. Yao, R. D. Hodge, A. M. Doyle, S. Jang, S. I. Shehata, A. M. Nelson, N. V. Shapovalova, B. P. Levi, S. Ramanathan, Fixed single-cell transcriptomic characterization of human radial glial diversity. *Nat. Methods* **13**, 87–93 (2016).

43. A. Pal, M. A. Noble, M. Morales, R. Pal, M. Baumgartner, J. W. Yang, K. M. Yim, S. Uebbing, J. P. Noonan, Resolving the three-dimensional interactome of human accelerated regions during human and chimpanzee neurodevelopment. *Cell* **188**, 1504–1523.e27 (2025).

44. I. S. Shimada, M. Acar, R. J. Burgess, Z. Zhao, S. J. Morrison, Prdm16 is required for the maintenance of neural stem cells in the postnatal forebrain and their differentiation into ependymal cells. *Genes Dev.* **31**, 1134–1146 (2017).

45. L. He, J. Jones, W. He, B. C. Bjork, J. Wen, Q. Dai, PRDM16 regulates a temporal transcriptional program to promote progression of cortical neural progenitors. *Development* **148**, dev194670 (2021).

46. W. A. Liu, S. Chen, Z. Li, C. H. Lee, G. Mirzaa, W. B. Dobyns, M. E. Ross, J. Zhang, S.-H. Shi, PARD3 dysfunction in conjunction with dynamic HIPPO signaling drives cortical enlargement with massive heterotopia. *Genes Dev.* **32**, 763–780 (2018).

47. M. Cayouette, M. Raff, Asymmetric segregation of Numb: A mechanism for neural specification from Drosophila to mammals. *Nat. Neurosci.* **5**, 1265–1269 (2002).

48. P. H. Petersen, K. Zou, J. K. Hwang, Y. N. Jan, W. Zhong, Progenitor cell maintenance requires numb and numblike during mouse neurogenesis. *Nature* **419**, 929–934 (2002).

49. W. Zhong, J. N. Feder, M. M. Jiang, L. Y. Jan, Y. N. Jan, Asymmetric localization of a mammalian numb homolog during mouse cortical neurogenesis. *Neuron* **17**, 43–53 (1996).

50. T. Heallen, M. Zhang, J. Wang, M. Bonilla-Claudio, E. Klysik, R. L. Johnson, J. F. Martin, Hippo pathway inhibits Wnt signaling to restrain cardiomyocyte proliferation and heart size. *Science* **332**, 458–461 (2011).

51. A. Reginensi, L. Enderle, A. Gregorieff, R. L. Johnson, J. L. Wrana, H. McNeill, A critical role for NF2 and the Hippo pathway in branching morphogenesis. *Nat. Commun.* **7**, 12309 (2016).

52. K.-P. Lee, J.-H. Lee, T.-S. Kim, T.-H. Kim, H.-D. Park, J.-S. Byun, M.-C. Kim, W.-I. Jeong, D. F. Calvisi, J.-M. Kim, D.-S. Lim, The Hippo-Salvador pathway restrains hepatic oval cell proliferation, liver size, and liver tumorigenesis. *Proc. Natl. Acad. Sci. U.S.A.* **107**, 8248–8253 (2010).

53. C. L. C. Poon, K. A. Mitchell, S. Kondo, L. Y. Cheng, K. F. Harvey, The hippo pathway regulates neuroblasts and brain size in Drosophila melanogaster. *Curr. Biol.* **26**, 1034–1042 (2016).

54. B. Zhao, X. Wei, W. Li, R. S. Udan, Q. Yang, J. Kim, J. Xie, T. Ikenoue, J. Yu, L. Li, P. Zheng, K. Ye, A. Chinnaiyan, G. Halder, Z.-C. Lai, K.-L. Guan, Inactivation of YAP oncoprotein by the Hippo pathway is involved in cell contact inhibition and tissue growth control. *Genes Dev.* **21**, 2747–2761 (2007).

55. A. Kawaguchi, Neuronal delamination and outer radial Glia generation in neocortical development. *Front. Cell Dev. Biol.* **8**, 623573 (2020).

56. D. Han, M. Kwon, S. M. Lee, S. J. Pleasure, K. Yoon, Non-cell autonomous promotion of astrogenesis at late embryonic stages by constitutive YAP activation. *Sci. Rep.* **10**, 7041 (2020).

57. J. H. Lui, T. J. Nowakowski, A. A. Pollen, A. Javaherian, A. R. Kriegstein, M. C. Oldham, Radial glia require PDGFD-PDGFRβ signalling in human but not mouse neocortex. *Nature* **515**, 264–268 (2014).

58. A. Uzquiano, A. J. Kedaigle, M. Pigoni, B. Paulsen, X. Adiconis, K. Kim, T. Faits, S. Nagaraja, N. Antón-Bolaños, C. Gerhardinger, A. Tucewicz, E. Murray, X. Jin, J. Buenrostro, F. Chen, S. Velasco, A. Regev, J. Z. Levin, P. Arlotta, Proper acquisition of cell class identity in organoids allows definition of fate specification programs of the human cerebral cortex. *Cell* **185**, 3770–3788.e27 (2022).

59. J. R. Soucy, L. Todd, E. Kriukov, M. Phay, V. V. Malechka, J. D. Rivera, T. A. Reh, P. Baranov, Controlling donor and newborn neuron migration and maturation in the eye through microenvironment engineering. *Proc. Natl. Acad. Sci. U.S.A.* **120**, e2302089120 (2023).

60. R. Balasubramanian, X. Min, P. M. J. Quinn, Q. L. Giudice, C. Tao, K. Polanco, N. Makrides, J. Peregrin, M. Bouaziz, Y. Mao, Q. Wang, B. L. Costa, D. Buenaventura, F. Wang, L. Ma, S. H. Tsang, P. J. Fabre, X. Zhang, Phase transition specified by a binary code patterns the vertebrate eye cup. *bioRxiv*. 2021.08.12.455556. (2021).

61. Y. Izumi, N. Ohta, K. Hisata, T. Raabe, F. Matsuzaki, Drosophila Pins-binding protein Mud regulates spindle-polarity coupling and centrosome organization. *Nat. Cell Biol.* **8**, 586–593 (2006).

62. Q. Du, P. T. Stukenberg, I. G. Macara, A mammalian Partner of inscuteable binds NuMA and regulates mitotic spindle organization. *Nat. Cell Biol.* **3**, 1069–1075 (2001).

63. T. M. Finegan, D. T. Bergstralh, Division orientation: Disentangling shape and mechanical forces. *Cell Cycle* **18**, 1187–1198 (2019).

64. O. Hertwig, *Das Problem Der Befruchtung Und Der Isotropie Des Eies: Eine Theorie Der Vererbung* (Verlag von Gustav Fischer, 1884).

65. C. B. O’Connell, Y.-L. Wang, Mammalian spindle orientation and position respond to changes in cell shape in a dynein-dependent fashion. *Mol. Biol. Cell* **11**, 1765–1774 (2000).

66. J. L. Fish, Y. Kosodo, W. Enard, S. Pääbo, W. B. Huttner, Aspm specifically maintains symmetric proliferative divisions of neuroepithelial cells. *Proc. Natl. Acad. Sci. U.S.A.* **103**, 10438–10443 (2006).

67. J. D. Godin, K. Colombo, M. Molina-Calavita, G. Keryer, D. Zala, B. C. Charrin, P. Dietrich, M.-L. Volvert, F. Guillemot, I. Dragatsis, Y. Bellaiche, F. Saudou, L. Nguyen, S. Humbert, Huntingtin is required for mitotic spindle orientation and mammalian neurogenesis. *Neuron* **67**, 392–406 (2010).

68. M. P. Postiglione, C. Jüschke, Y. Xie, G. A. Haas, C. Charalambous, J. A. Knoblich, Mouse inscuteable induces apical-basal spindle orientation to facilitate intermediate progenitor generation in the developing neocortex. *Neuron* **72**, 269–284 (2011).

69. Y. Zhou, H. Song, G.-L. Ming, Genetics of human brain development. *Nat. Rev. Genet.* **25**, 26–45 (2024).

70. C. Llinares-Benadero, V. Borrell, Deconstructing cortical folding: Genetic, cellular and mechanical determinants. *Nat. Rev. Neurosci.* **20**, 161–176 (2019).

71. B. K. Terry, S. Kim, The role of Hippo-YAP/TAZ signaling in brain development. *Dev. Dyn.* **251**, 1644–1665 (2022).

72. G. B. Collin, J. Won, M. P. Krebs, W. J. Hicks, J. R. Charette, J. K. Naggert, P. M. Nishina, Disruption in murine Eml1 perturbs retinal lamination during early development. *Sci. Rep.* **10**, 5647 (2020).

73. B. Tarchini, C. Jolicoeur, M. Cayouette, A molecular blueprint at the apical surface establishes planar asymmetry in cochlear hair cells. *Dev. Cell* **27**, 88–102 (2013).

74. T. Murata, K. Furushima, M. Hirano, H. Kiyonari, M. Nakamura, Y. Suda, S. Aizawa, Ang is a novel gene expressed in early neuroectoderm, but its null mutant exhibits no obvious phenotype. *Gene Expr. Patterns* **5**, 171–178 (2004).

75. A. F. Tarantal, "Ultrasound imaging in rhesus (Macaca mulatta) and long-tailed (Macaca fascicularis) macaques: Reproductive and research applications" in *The Laboratory Primate* (Elsevier, 2005), pp. 317–352.

76. F. A. Wolf, P. Angerer, F. J. Theis, SCANPY: Large-scale single-cell gene expression data analysis. *Genome Biol.* **19**, 15 (2018).

77. A. Javed, P. Mattar, S. Lu, K. Kruczek, M. Kloc, A. Gonzalez-Cordero, R. Bremner, R. R. Ali, M. Cayouette, Pou2f1 and Pou2f2 cooperate to control the timing of cone photoreceptor production in the developing mouse retina. *Development* **147**, dev188730 (2020).

78. M. Langouët, C. Jolicoeur, A. Javed, P. Mattar, M. D. Gearhart, S. P. Daiger, M. Bertelsen, L. Tranebjærg, N. D. Rendtorff, K. Grønskov, C. Jespersgaard, R. Chen, Z. Sun, H. Li, N. Alirezaie, J. Majewski, V. J. Bardwell, R. Sui, R. K. Koenekoop, M. Cayouette, Mutations in BCOR, a co-repressor of CRX/OTX2, are associated with early-onset retinal degeneration. *Sci. Adv.* **8**, eabh2868 (2022).

79. D. Legland, I. Arganda-Carreras, P. Andrey, MorphoLibJ: Integrated library and plugins for mathematical morphology with ImageJ. *Bioinformatics* **32**, 3532–3534 (2016).

80. U. Schmidt, M. Weigert, C. Broaddus, G. Myers, "Cell detection with star-convex polygons" in *Medical Image Computing and Computer Assisted Intervention – MICCAI 2018* (Springer International Publishing, Cham, 2018) *Lecture notes in computer science*, pp. 265–273.

81. D. J. Di Bella, E. Habibi, R. R. Stickels, G. Scalia, J. Brown, P. Yadollahpour, S. M. Yang, C. Abbate, T. Biancalani, E. Z. Macosko, F. Chen, A. Regev, P. Arlotta, Molecular logic of cellular diversification in the mouse cerebral cortex. *Nature* **595**, 554–559 (2021).
